# Impact of Local Structure in Supported CaO Catalysts for Soft-Oxidant-Assisted Methane Coupling Assessed through Ca K-Edge X-ray Absorption Spectroscopy

**DOI:** 10.1021/acs.jpcc.3c06527

**Published:** 2024-01-10

**Authors:** Leah R. Filardi, Fernando D. Vila, Jiyun Hong, Adam S. Hoffman, Jorge E. Perez-Aguilar, Simon R. Bare, Ron C. Runnebaum, Coleman X. Kronawitter

**Affiliations:** † Department of Chemical Engineering, University of California, Davis, Davis, California 95616, United States; ‡ Department of Physics, University of Washington, Seattle, Washington 98195, United States; § SSRL, SLAC National Accelerator Laboratory, Menlo Park, California 94025, United States; ∥ Department of Viticulture & Enology, University of California, Davis, Davis, California 95616, United States

## Abstract

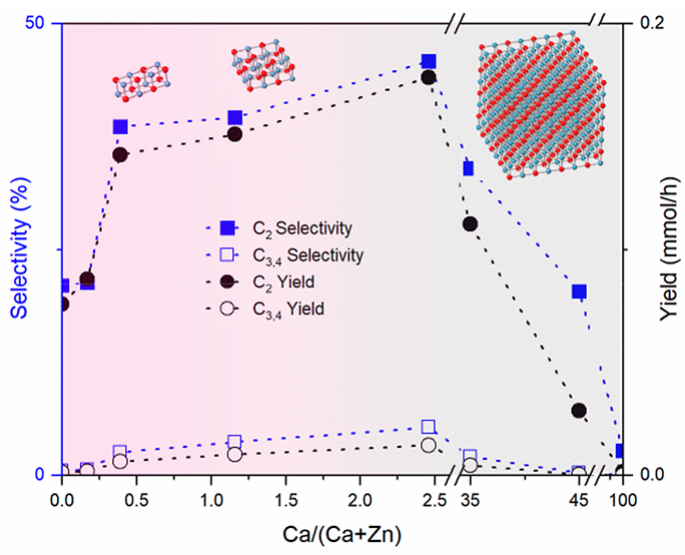

Soft-oxidant-assisted methane coupling has emerged as a promising pathway to upgrade methane from natural gas sources to high-value commodity chemicals, such as ethylene, at selectivities higher than those associated with oxidative (O_2_) methane coupling (OCM). To date, few studies have reported investigations into the electronic structure and the microscopic physical structure of catalytic active sites present in the binary metal oxide catalyst systems that are known to be effective for this reaction. Correlating the catalyst activity to specific active site structures and electronic properties is an essential aspect of catalyst design. Here, we used X-ray absorption spectroscopy at the Ca K-edge to ascertain the most probable local environment of Ca in the ZnO-supported Ca oxide catalysts. These catalysts are shown here to be active for N_2_O-assisted methane coupling (N_2_O-OCM) and have previously been reported to be active for CO_2_-assisted methane coupling (CO_2_-OCM). X-ray absorption near edge structure features at multiple Ca loadings are interpreted through simulated spectra derived from *ab initio* full multiple scattering calculations. These simulations included consideration of CaO structures organized in multiple spatial arrangements—linear, planar, and cubic—with separate analyses of Ca atoms in the surfaces and bulk of the three-dimensional structures. The morphology of the oxide clusters was found to influence the various regions of the X-ray absorption spectrum differently. Experiment and theory show that for low-Ca-loading catalysts (≤1 mol %), which contain sites particularly active for methane coupling, Ca primarily exists in an oxidized state that is consistent with the coordination environment of Ca ions in one- and two-dimensional clusters. In addition to their unique nanoscale structures, the spectra also indicate that these clusters have varying degrees of undercoordinated surface Ca atoms that could further influence their catalytic activities. The local Ca structure was correlated to methane coupling activity from N_2_O-OCM and previously reported CO_2_-OCM reactor studies. This study provides a unique perspective on the relationship between the catalyst physical and electronic structure and active sites for soft-oxidant-assisted methane coupling, which can be used to inform future catalyst development.

## 1 Introduction

There has been a recent surge in natural gas development, and the U.S. Department of Energy expects continual growth for at least the next several decades.^1^ Natural gas is often a byproduct of oil extraction^2^ in low-population areas which poses a major economic challenge for gas transportation.^3^ As a result, it is often flared into carbon dioxide or released, emitting greenhouse gases to the atmosphere.^2^ Roughly 90% of the end-use of natural gas is combustion for heat or energy, further adding carbon dioxide to the atmosphere.^4^ Upgrading methane, the dominant chemical species present in natural gas, into a more energy-dense chemical or fuel would allow for a more efficient utilization of this abundant resource and help to reduce greenhouse gas emissions.

Oxidative coupling of methane (OCM) is a highly explored avenue of methane upgrading to larger hydrocarbons via C–C bond formation.^5^ However, the presence of strong oxidant species at high temperatures leads to a strong trade-off between methane conversion and C_2_ product selectivity^6^ where undesirable combustion reactions of methane and C_2_ products limit product yields below 30%.^7^ However, the use of softer oxidants that do not form gas-phase oxygen species offers an opportunity to break the trade-off relationship. Carbon dioxide and nitrous oxide are both emerging oxidants to replace oxygen during OCM.^8−11^ C_2_ product selectivity over 80% can easily be achieved with binary metal oxide catalysts, notably when coupling a basic oxide with a reducible oxide.^12−14^

This cooperation has been demonstrated by previous efforts to characterize these binary oxide catalysts. For many bulk reducible metal oxides including MnO_2_,^15^ CeO_2_,^14^ CoO, CuO, Bi_2_O_3_, and Fe_2_O_3_,^16^ XRD and XPS^17^ have demonstrated their partial reduction after reaction. Reaction in the absence of any oxidant leads to complete reduction,^13^ indicating CO_2_- and N_2_O-OCM proceed through a Mars–van Krevelen mechanism where oxygen vacancies are formed during the reaction with methane and are replenished by the oxidant.^12^ The presence of oxygen vacancies has been confirmed in PrO_2_ via O_2_-TPD.^18^ Addition of a basic metal oxide can promote CO_2_ chemisorption for increased conversion over the reducible sites during CO_2_-OCM, demonstrated by CO_2_-TPD.^12,19^ Catalyst basicity and reducibility were also shown to correlate with C_2_ product yields for N_2_O-OCM.^20^

Recent work used CO_2_-IR-TPD to establish a volcano relationship between catalyst basicity and CO_2_-OCM performance.^21^ The interface between basic and reducible metal oxides in a Ca/ZnO catalyst had the presence of medium-strength basicity and was proposed to be responsible for selective methane coupling. It was also observed that very low concentrations of calcium (∼0.6 mol %) exhibited excellent catalytic performance, and the Ca sites had electronic and geometric properties that were not characteristic of bulk CaO species, according to various characterization methods, including Ca L-edge X-ray absorption near edge structure (XANES). However, the specific nature of these Ca species has not been thoroughly investigated.

X-ray absorption spectroscopy (XAS) is a useful tool for determining the local structure of metal oxide nanoparticles. Decreases in second and third shell coordination numbers, relative to bulk metal oxide, have been correlated to nanoparticles of defined sizes.^22,23^ These results in tandem with DFT calculations have predicted specific structures of supported metal oxide clusters with 9–13 metal atoms.^24^ There have been few studies that use Ca K-edge extended X-ray absorption fine structure (EXAFS) to inform on Ca-containing nanoparticle sizes and structure of highly dispersed^25,26^ and nanoparticle Ca species.^27,28^ Changes in Ca–O coordination can be observed during mineral crystallization as the solid phases become larger and more ordered.^29^ Ca deposition on coal via impregnation and ion exchange yielded catalysts that lacked any Ca–Ca coordination due to a high degree of dispersion.^25^ The aggregation of CaCO_3_ and CaO species was observed after high-temperature treatments.

This work uses Ca K-edge XAS paired with theoretical XAS simulations to evaluate the local coordination environment of Ca species in Ca/ZnO catalysts over the wide range of Ca compositions previously studied^21^ and correlate structure to CO_2_-OCM and N_2_O-OCM performance. The structure of low-Ca-loading catalysts is contrasted with that of bulk CaO species to reveal the presence of ultrasmall CaO clusters with one-dimensional linear and two-dimensional planar geometries. The difference in active site structure is associated with enhanced methane coupling performance for low-Ca-loading Ca/ZnO catalysts.

## 2 Materials and Methods

### 2.1 Catalyst Synthesis

Calcium carbonate (98% purity) and calcium hydroxide (98% purity) were acquired from VWR and Acros Organics, respectively. Calcium oxide–zinc oxide composite catalysts were prepared via a wet impregnation synthesis as described earlier.^21^ Briefly, calcium nitrate tetrahydrate (99%, ACS Reagent, MP Biomedicals) was dissolved in Milli-Q (18 Mohm) water. Zinc oxide (99.9% metal basis, Alfa Aesar) was added, and the resulting slurry was sonicated and stirred partially covered overnight. The resulting white solids were dried at 120 °C for 24 h before being calcined in 50 mL/min air (zero air, Praxair) at 850 °C (ramp 5 °C/min) for 4 h. Henceforth, the Ca loading will be denoted by the resulting Ca/(Zn + Ca) ratio, or mol % of cation. Calcium oxide was synthesized by heating 100 mL of a 0.25 M calcium nitrate solution in an oil bath to 80 °C.^30^ Under vigorous stirring, a 50 mL solution of 1 M NaOH was added dropwise, resulting in a cloudy solution of a white precipitate. The solids were vacuum filtered, washed with deionized water, and dried overnight at 120 °C before calcination (see above). Calcium weight loading was determined by ICP-OES (Table S2).

### 2.2 Materials Characterization

Calcium K-edge XAS data were collected at beamline 4-3 of the Stanford Synchrotron Radiation Lightsource equipped with a double-crystal Si(111) monochromator at an orientation of Φ = 90°. Helium was used as the ionization chamber gas with the ion chamber voltage at 200 V DC and negative polarity. To minimize absorption by air at these low photon energies, a UHP helium-purged tube was installed between the first ion chamber and the sample stage. The photon energy was initially calibrated with a Ti metal foil in transmission mode. The beamline was calibrated by defining the edge energy, the maximum if the first derivative of the Ti foil spectra, to 4966.0 eV. Subsequently, an initial reference spectrum of CaCO_3_ was acquired where *E*_0_ was observed to be 4037.6 eV. Measurements of CaCO_3_ were repeated at least every 24 h, and *E*_0_ was aligned to 4037.6 eV to account for any drift in the monochromator. Spectra were acquired between 3805.0 and 4645.0 eV. At least 4 spectra of each sample were acquired and merged to improve the signal-to-noise ratio. All data were collected in fluorescence mode. For calcium catalysts with ≤2 mol %, data were collected using a 7-element Canberra silicon-drift detector. For all other samples and standards, a PIPS detector was used. Approximately 1–3 mg of sample was ground to a fine powder and smeared as a thin uniform layer onto a zero-sulfur-containing adhesive tape and placed in an airtight cell inside a glovebox and then transferred to the beamline stage with helium flow.

Post-processing and analysis of the XAS data were performed using the Athena and Artemis software of the Demeter package.^31^ Energy correction was applied from the corresponding CaCO_3_ reference spectrum. The pre-edge region in the range of 43.4–20.0 eV below the edge was normalized with a linear fit and in the range of 50.0–400.0 eV above the edge with a second-order polynomial fit. The data were modeled in *q*-space to reduce any errors associated with fitted coordination numbers by filtering out high-frequency (scattering paths at longer *R*) components from the data. The data were fit in the *q* range of 3.4–9.1 Å^–1^_._ The same *k* range and a back-Fourier transform *R* range of 1.0–3.5 Å was used. EXAFS models were built using scattering paths generated from CIF files using FEFF6. CIF files were downloaded from the Materials Project^32^ and consisted of Ca(OH)_2_ (mp-23879), CaO (mp-2605), and ZnO (mp-2133) where the central Zn atom was replaced with a Ca atom to simulate an isolated Ca atom within the ZnO lattice. The amplitude reduction factor *S*_0_^2^ was determined from a fit of 2 mol % Ca/ZnO to be 0.73. For all catalysts, the Ca–O coordination number was fixed to 6. The continuous Cauchy wavelet transform (CCWT) analysis on the EXAFS data was done using Python codes based on Larch modules.^33^ The analysis was performed on the *k* range of 3.4–9.1 Å^–1^ for CaO, 1 mol % Ca/ZnO, and 0.6 mol % Ca/ZnO EXAFS spectra_._ The *k*^2^-weighted CCWT was calculated for the *k* range of 0–9.1 Å^–1^ and *R* range of 0.2–6.0 Å.

### 2.3 Computational Details

The XANES simulations were performed with FEFF10.^34−36^ The SCF stage used a cutoff radius of 5 Å and maximum angular momenta of 3 and 2 for Ca and O, respectively. The FMS stage used a cutoff radius of 9 Å, with maximum angular momenta of 4 and 3 for Ca and O, respectively. These values ensured convergence for the bulk case. The model for the bulk CaO simulations was generated from the experimental structure^37^ of CaO with space group *Fm*3*m* and lattice constant 4.8105 Å, resulting in a Ca–O bond distance of 2.405 Å. The model for Ca in the surface was generated by cleaving the bulk model in order to expose the (100) surface. In order to explore the effect on the XANES of a broad range of sizes, morphologies, and local environments for the CaO, which could vary as a function of loading, unsupported cluster models were generated by repeating the neutral Ca_4_O_4_ cubic unit along one, two, or three directions in order to generate “linear”, “planar”, and “cubic” clusters (as shown in the insets of Figure 4). These are labeled as (*n*,*l*,*m*) according to the number of cubic units in each direction. Based on the Ca–O and Ca–Ca bond distances obtained in the EXAFS fits and exploratory supported relaxations (see below), all of the model cluster structures retain the original, unrelaxed crystal structure. All calculations used the final state rule approximation for the core–hole and the default Hedin–Lundqvist approximation for the self-energy. The calculations also included room temperature thermal damping at the level of single scattering, using the correlated Debye model with a Debye temperature of 562 K. This temperature was chosen to match the fitted σ^2^ for the Ca–O paths in the catalysts at 300 K. While the bulk and surface simulations were performed on a single representative Ca atom, for the (*n*,*l*,*m*) clusters the XANES calculations were performed for each Ca atom in the structure, and the resulting spectra were averaged to obtain the final result. The effect of dipole-forbidden transitions was studied by adding the MULTIPOLE card to the FEFF calculations to include the quadrupole contributions. In addition, the effect of s → d transitions were quantified by removing the s → p contributions from the total spectra. The theoretical spectra were normalized using a procedure similar to that used in EXAFS analysis in which the post-edge decay of the atomic background is removed. The difference between the XANES of the catalysts and that of bulk CaO (ΔXANES) was quantitatively compared to that of the (*n*,*l*,*m*) clusters and the theoretically simulated bulk CaO. This was done using a Frechet form figure of merit (FOM), described in detail in the Supporting Information. Finally, in order to investigate the suitability of unrelaxed, unsupported cluster models for the assessment of the changes in the XANES as a function of loading, a ZnO-supported CaO model was also constructed by creating an epitaxial contact^38^ between the O surface of the (0001) plane of ZnO and the equivalent surface of the (111) plane in CaO, with the latter being contracted by 4.5% to match the ZnO surface.^37^ From this structure, a pyramidal CaO cluster of side ∼9 Å was carved to expose the (100) surfaces. The internal degrees of freedom (excluding the bottom two layers of ZnO support) of the resulting simulation cell (*a* = *b* = 19.497 Å, *c* = 25.0 Å, α = β = 90.0°, γ = 120.0°) were optimized with VASP using the PBE functional, a plane-wave cutoff of 400 eV, and PAW pseudopotentials (Figure S9). The supported structure expands laterally to release virtually all of the epitaxial contraction, resulting in an average bond distance of 3.40 Å, compared to the bulk CaO distance of 3.39 Å at this level of theory. This results in a bond strain index of 1.00 ± 0.01. The Ca–Ca scattering length distribution is narrow, with a static mean-square relative displacement (MSRD) of 3.6 × 10^–3^ Å^2^. The Ca–Zn shell at 3.43 Å is highly disordered with a MSRD of 22.4 × 10^–3^ Å^2^, which will be relevant to later discussions. The structural similarities between this supported, relaxed example and the rigid models described above justify the latter use in the general XANES simulations.

### 2.4 Catalytic Activity Measurements

N_2_O-OCM experiments over a series of Ca loadings were conducted in a quartz downflow, packed-bed reactor with a 4 mm ID. A sample mass of 0.750 g was sieved between 250 and 425 μm and loaded into the tube, supported by quartz wool (Acros Organics). The reactor was heated in a tubular furnace at 5 °C min^–1^ to 800 °C under 13.3 mL min^–1^ argon (99.997%, Praxair) and then quickly cooled to 550 °C. Flow was then switched to bypass the reactor and adjusted to 3.3 mL min^–1^ methane (99.9995%, Matheson), 6.7 mL min^–1^ nitrous oxide (99.998%, Linde), and 3.3 mL min^–1^ argon prior to flow through the reactor. Reaction products were measured by an online Agilent 7890A gas chromatograph (GC) with a CP-SilicaPLOT column to quantify hydrocarbon species with FID and Porapak Q and ShinCarbon ST columns to quantify all other species with TCD. Separate N_2_O-OCM experiments were performed as follows to determine activation energies. Activation energies were determined after verification that the reactor was in a differential regime. A sample mass of 0.2 g and a total flow rate of 30 mL min^–1^ with varying feed ratios were used. The reactor was heated in a tubular furnace at 5 °C min^–1^ to 800 °C under 13.3 mL min^–1^ argon and then quickly cooled to 520 °C. Flow was then switched to bypass the reactor and stabilize for 45 min. The reactor temperature was held for 2.5 h while injecting to the GC, then quickly ramped by 10 °C, and held for another 2.5 h. This proceeded from 520 to 560 °C. Steady-state data over the 2.5 h period were averaged.

## 3 Results and Discussion

### 3.1 Evaluation of Ca K-Edge XANES and Pre-Edge Structure

**Figure 1:**
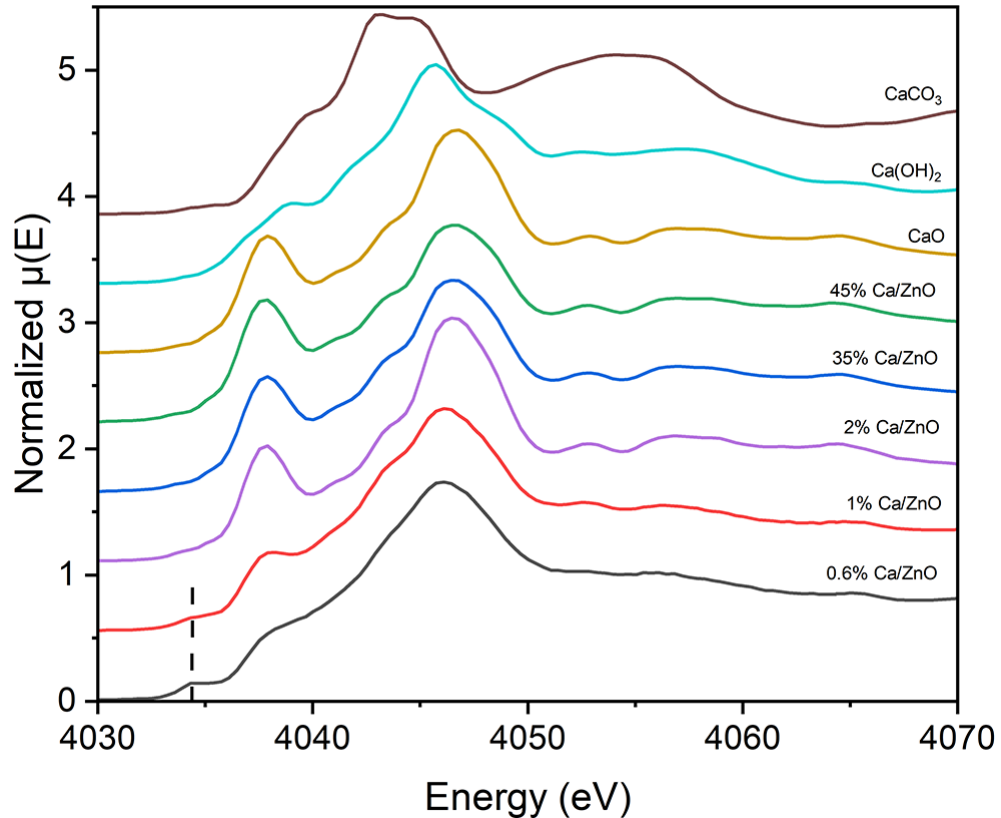
*Ex situ* Ca K-edge XANES spectra were collected at room temperature in He. The dashed line is to visualize the pre-edge feature. Spectra are offset for clarity.

The XANES spectra of Ca/ZnO catalysts with varying concentrations of Ca are compared to common bulk Ca reference compounds—CaO, Ca(OH)_2_, and CaCO_3_ in Figure 1. The overall shape of the spectra of all Ca/ZnO catalysts resembles that of CaO, with the white line absorption centered at 4046.8 eV and a significant shoulder feature at 4037.9 eV. Spectra of catalysts with 2 mol % and higher Ca content are nearly identical with that of CaO, indicating that Ca exists in its bulk oxide phase in these higher Ca-loaded catalysts (Figure S1). This is consistent with XRD, TEM, and L-edge XANES results, published in prior work.^21^ Below 2 mol % Ca content, the shoulder feature diminishes, the white line peak decreases and broadens, a new pre-edge feature grows at 4034.3 eV, and post-edge oscillations are significantly dampened. These features resemble those seen in the spectrum of Ca(OH)_2_, but rigorous EXAFS analysis verifies the absence of a hydroxide phase; XAS associated with the hydroxide phase is addressed later. The new pre-edge feature is present only in the two lowest Ca-loading catalysts. Several different physical assignments of this peak have been reported.^39,40^ Theoretical simulations of Ca local density of states, provided later in this report, provide justification for assigning this peak to a Ca 1s to p electronic transition.

### 3.2 Characterization of CaO Structure by EXAFS Analysis

Modeling of the EXAFS spectra provides information on the local structure of dispersed Ca sites present in the low-Ca-loading catalysts—1 and 0.6 mol % Ca/ZnO. To determine the precise Ca coordination environment, a *q*-space fit was used^41^ to filter out high-frequency components to quantify observed changes in specifically the first two scattering paths (see the Methods section for more details). At high Ca composition, self-absorption^42^ distorts the data for >2 mol % Ca, which prevents EXAFS analysis of those catalysts. The 2 mol % Ca/ZnO catalyst was reported to contain CaO particles >40 nm in size, determined by XRD.^21^ This sample has well-defined XAS features that demonstrate the absence of the self-absorption effects observed with higher Ca loadings. Combined, these results reinforce our assumption that the Ca speciation in 2 mol % Ca/ZnO catalyst is equivalent to that of bulk CaO. Thus, the 2 mol % Ca/ZnO catalyst was used to determine the value of *S*_0_^2^, 0.73 (Figure S2 and Table S3), which is consistent with a previously reported value of 0.8.^43^

**Figure 2:**
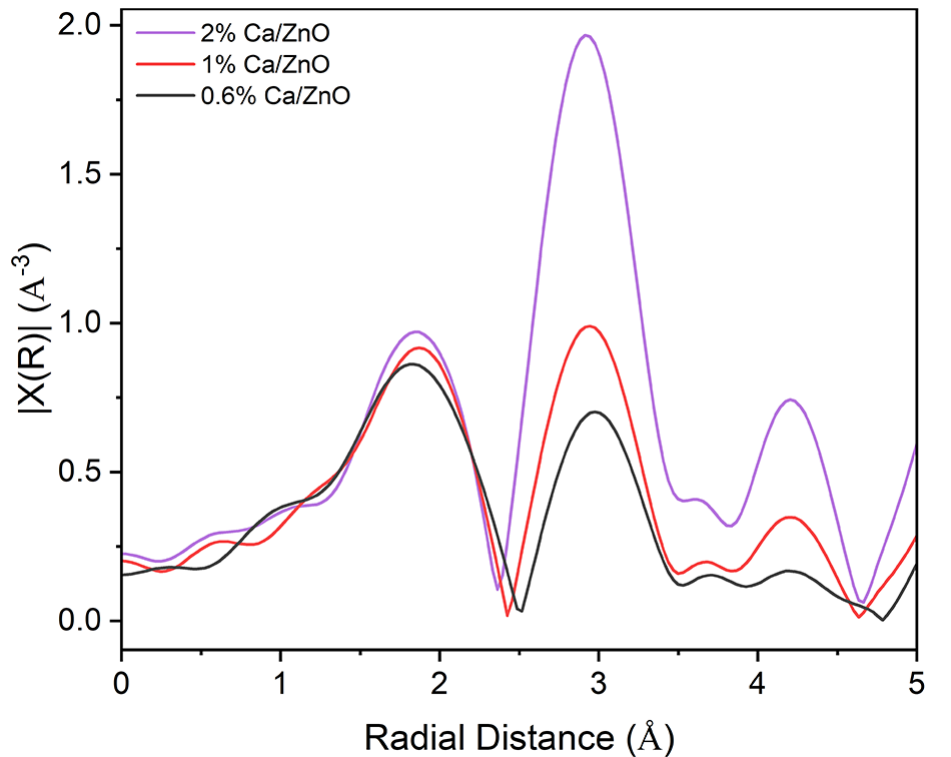
Magnitudes of the Fourier transforms of phase-uncorrected *k*^2^-weighted EXAFS of low-Ca-loading Ca/ZnO catalysts using the *k* range of 3.4–9.1 Å^–1^.

The magnitudes of the Fourier transforms of the EXAFS spectra for the low-Ca-loading catalysts are shown in Figure 2. The first peak is comparable in intensity across the three catalysts. The 1 and 0.6 mol % Ca/ZnO catalysts exhibit a significant decrease in all peak intensities at distances beyond 2.4 Å. Path analysis of CaO (Figure S3) shows that the first peak is associated with Ca–O at a bonding distance of 2.42 Å and the second peak is only associated with Ca–Ca single scattering at a distance of 3.42 Å. These results suggests that at loadings ≤1 mol % the Ca oxide exists as nanoparticles significantly smaller than those in the higher-Ca-loading catalysts; we henceforth refer to the ultrasmall Ca oxide structures in the two low-Ca-loading catalysts as clusters.

**Figure 3:**
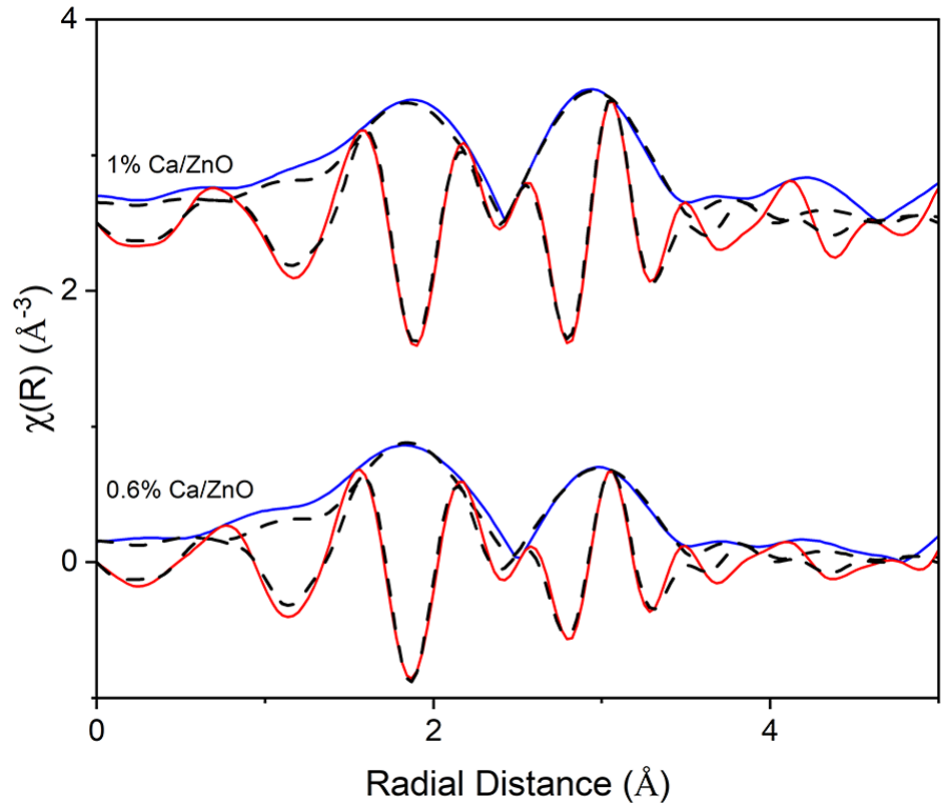
Magnitudes and imaginary portions of the *k*^2^-weighted *R*-space functions for spectra associated with the low-Ca-loading catalysts are shown using solid blue and red lines, respectively; fits are shown with dashed lines. The data were back-Fourier transformed in the *R* range of 1.0–3.5 Å and fit in the *q* range of 3.4–9.1 Å^–1^.

The two low-Ca-loading catalysts were modeled with the Ca–O and Ca–Ca scattering paths of bulk CaO. The Ca–O coordination was first allowed to float but always converged to a value close to 6. In subsequent models, it was fixed to 6 to simplify the model. This is a safe assumption because the first shell peak intensity in *R*-space is equivalent to the 2 mol % Ca/ZnO reference, and isolated Ca atoms have been reported to maintain a bulk Ca–O coordination number for the nearest-neighbor coordination.^25^ The fitting results are listed in Figure 3 and Table 1. The data of the high-Ca-loading catalysts are presented in the *R*-, *q*-, and *k*-spaces in Figure S4 without fits. All fits result in oxygen and calcium distances associated with bulk CaO with little to no increases in the σ^2^. The lack of difference in the Ca–O bond length and degree of disorder supports a similar coordination number to that of bulk CaO.^44^ This is consistent with reports that Ca nanostructures undergo negligible lattice distortion and maintain rigidity.^45,46^ The Ca–Ca coordination numbers are 5.9 ± 0.4 and 4.1 ± 0.7 for the 1 and 0.6 mol % Ca/ZnO catalysts, respectively. These values are significantly smaller than the bulk CaO Ca–Ca coordination number of 12, indicating that calcium exists as nanoparticles of decreasing size.^22,47^ Structural disorder could also lead to a reduction of long-range coordination but would predominately influence the third and higher shells.^22^ The Ca–O and Ca–Ca scattering distances remain within error of those of bulk CaO, indicating that little to no CaO lattice distortion occurs with a decreasing cluster size. This finding simplifies the theoretical modeling by allowing for the use of unrelaxed structures in the XANES simulations.

**Table 1:** Best Fit of the EXAFS Parameters for Ca/ZnO Catalysts^a^

	path	CN	*R* (Å)	σ^2^ × 10^3^ (Å^2^)	Δ*E*_0_ (eV)	*R*-factor	*S*_0_^2^
1% Ca/ZnO	Ca–O	6	2.37 ± 0.01	6.1 ± 1.6	0.8 ± 1.0	0.0111	0.73
	Ca–Ca	5.9 ± 0.4	3.41 ± 0.01	4.6			0.73
0.6% Ca/ZnO	Ca–O	6	2.36 ± 0.02	6.7 ± 1.9	1.6 ± 1.8	0.0347	0.73
	Ca–Ca	4.1 ± 0.7	3.42 ± 0.03	4.6			0.73
Simulated CaO clusters	Ca–O^b^		2.41				
	Ca–Ca^c^		3.40				

a Notation: CN, coordination number; *S*_0_^2^, amplitude correction term; Δ*E*_0_, energy correction factor; *R*, scattering path length; σ^2^, disorder term. A *k* range of 3.4–9.1 Å^–1^ and an *R* range of 1.0–3.5 Å were used. Values without error bounds were held constant.

b The CN of the Ca–O scattering paths cannot be directly compared to experimental results because the Ca atoms on the surface of the simulated structures were uncapped rather than oxygen-terminated.

c The CN of the Ca–Ca scattering paths range from 4.0–4.7, 5.3–7.0, and 6.8–9.2 for the linear, planar, and cubic theoretical clusters, respectively.

A Ca–Zn scattering path cannot be quantifiably modeled from the EXAFS data. While the data can be fit with a Ca–Zn scattering path, the resulting σ^2^ and Δ*E*_0_ values are unrealistically large (Figures S5, S6 and Tables S4, S5). Figure S7 shows the simulated *k*-space oscillations of Ca–Ca and Ca–Zn scattering paths compared to the experimental spectrum of 0.6 mol % Ca/ZnO. The amplitudes of the oscillations from the Ca–Zn scattering path peak at ∼10 Å^–1^ where the amplitudes of the experimental data approaches zero. However, the convolution of these spectra of paths with similar distances (3.4 Å) reveals a conclusive determination of the influence of the Ca–Zn scattering path on the experimental data. Continuous Cauchy wavelet transform (CCWT) analysis of the EXAFS spectra was performed to deconvolute the *R*-space peak at 3 Å. CCWT analysis demonstrates that there is no difference in the *k*-space oscillation intensity between 1 and 0.6 mol % Ca/ZnO compared to pure CaO (Figure S8). Therefore, there is no detectable scattering from a heavier element. Exploratory simulations of the relaxed CaO cluster supported by ZnO yield an average Ca–Zn distance of 3.43 Å, a coordination number of 3.0, and a static mean-squared relative displacement of 22.4 × 10^–3^ Å^2^ (Figure S9). The influence of the ZnO is therefore difficult to observe despite its known presence due to both the low Ca–Zn coordination number and high degree of static disorder at a similar distance to the Ca–Ca interaction. Thus, models with Ca–Zn scattering paths have not been further analyzed. Additionally, models of calcium hydroxide or mixtures of oxide and hydroxide were ruled out due to poor fitting of the data with large *R*-factor, σ^2^, and Δ*E*_0_ values (Figures S10, S11 and Tables S6, S7).

### 3.3 Computational Simulation of X-ray Absorption Spectra of CaO Clusters

**Figure 4:**
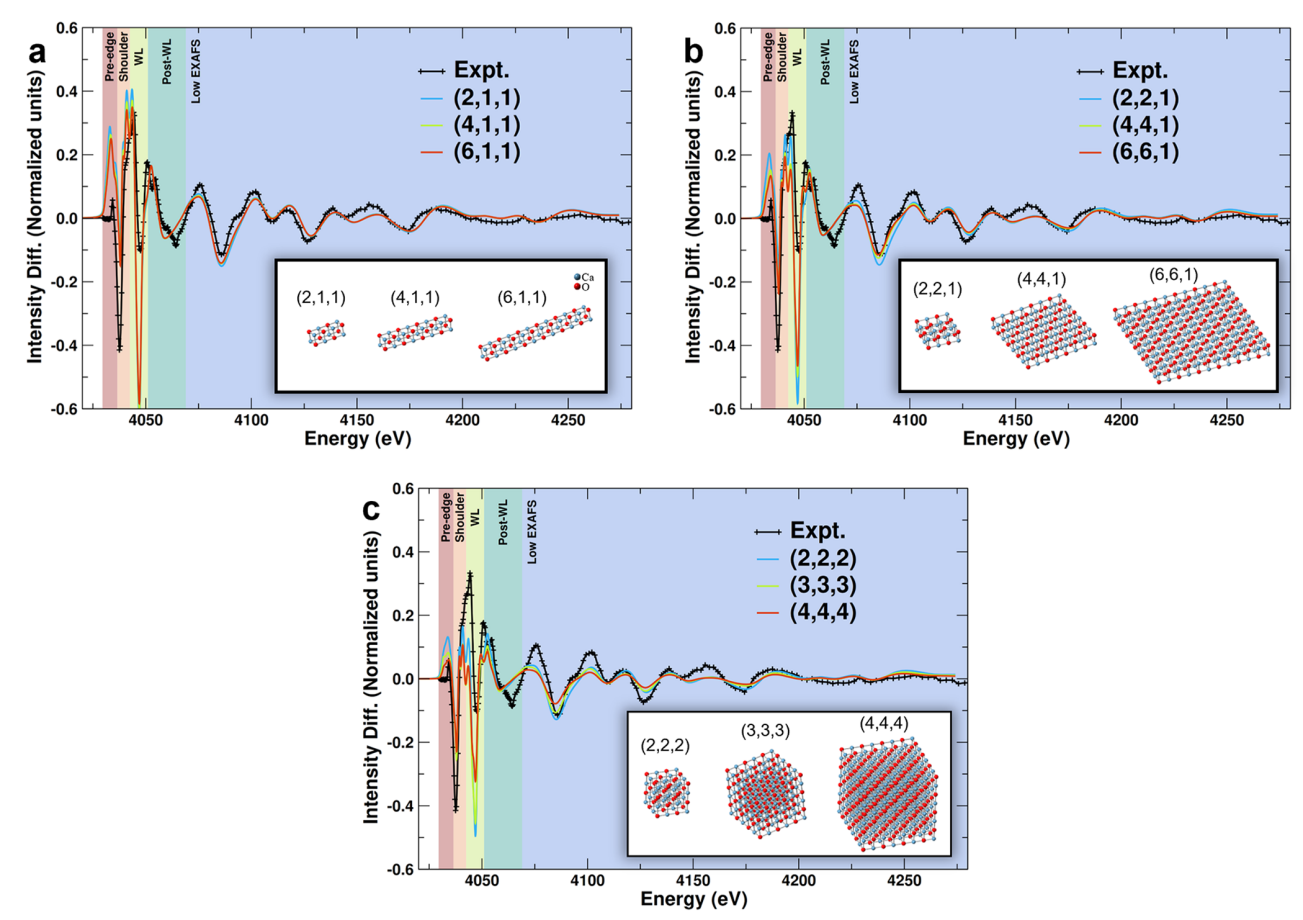
Difference between the experimental spectra of 0.6 mol % Ca/ZnO and CaO reference compared to the difference between simulated spectra of CaO nanoparticles of varying sizes and bulk CaO for the (a) linear, (b) planar, and (c) cubic morphologies. The numbers in parentheses represent the number of Ca_4_O_4_ units in each dimension. The CN of the Ca–Ca scattering path ranges from 4.0–4.7, 5.3–7.0, and 6.8–9.2 for the linear, planar, and cubic theoretical clusters, respectively.

Theoretical simulations of XANES spectra provide additional complementary insight into the specific structures responsible for spectral features observed. The XANES spectra associated with the Ca/ZnO catalysts were simulated using FEFF10, as described in the Methods section. These simulations included consideration of CaO structures organized in multiple spatial arrangements, with separate analyses of Ca atoms in the surface and bulk of three geometries—linear, planar, and cubic—shown in insets of Figure 4. The spectrum of bulk CaO was simulated using a Ca atom surrounded by 6 and 7 full coordination layers for both O and Ca, respectively. This system was used to find the best computational parameters for FEFF and to assess the quality of the XANES simulations for a system with a known structure. The spectrum contains all major features found in the experimental spectrum (Figure S12). Using those parameters, the XANES of a Ca atom on the (100) surface of CaO was also calculated (Figure S13) and found to qualitatively reproduce all the characteristic differing features in the spectrum for a low-Ca-loading catalyst versus bulk CaO—decreased intensity of the shoulder, white line, and EXAFS oscillations as well as increased intensity of a new pre-edge peak. The agreement between the spectrum for 0.6 mol % Ca/ZnO and that simulated for surface atoms supports the existence of clusters with a high fraction of exposed and undercoordinated Ca atoms. Computational investigations into the growth of (CaO)_*n*_ clusters found that cubic-like structures, including linear and planar geometries, were most favorable and stable.^46,48,49^ Additionally, the experimentally observed Ca–O and Ca–Ca scattering distances of the 0.6 and 1 mol % Ca/ZnO catalysts are consistent with those of bulk CaO. Therefore, unrelaxed clusters with a range of sizes and Ca–Ca coordination numbers were modeled in linear, planar, and cubic geometries (Figure S14) using bulk Ca–O and Ca–Ca distances of 2.41 and 3.40 Å, respectively, as shown in Table 1. The unrelaxed structures provide a convenient, sound approximation because of the negligible differences in the Ca–O bond distance and σ^2^ between the experimental bulk CaO and low-Ca-loading EXAFS fits. The experimentally determined Ca–Ca coordination numbers were 4.1 ± 0.7 and 5.9 ± 0.4 for 0.6 mol % Ca/ZnO and 1 mol % Ca/ZnO catalysts, respectively, which best correspond to linear and planar structures.

Figure S15 shows the average theoretical spectra for each of these structures compared to bulk CaO. Because the main objective is to determine the origin of differences between the low-Ca-loading systems and bulk CaO, the difference spectrum (ΔXANES) between the 0.6 mol % Ca/ZnO catalyst and bulk CaO was generated—it provides a direct visualization of differences in each spectral region. This difference in experimental results was compared to the differences between the theoretical XANES of the linear, planar, and cubic CaO clusters and that of bulk CaO. To provide a more quantitative measure of agreement between theory and experiment, a normalized Frechet distance was calculated as a FOM (Table S8) for the complete theoretical energy range as well as individual ranges that characterize energy regions of importance—pre-edge, shoulder, white line, post-edge, and low EXAFS. The baseline accuracy of the theoretical spectra to the experimental spectra was assessed by applying the Frechet analysis to the experimental versus theoretical CaO bulk data. We find that the largest deviations between theory and experiment (Table S8, bottom) occur in the shoulder and white line regions of the spectra. This is likely due to the use of the muffin-tin potential approximation in FEFF. Therefore, these regions should be considered less relevant when the ΔXANES results are analyzed.

Visual inspection of the ΔXANES results shows the spectra generated from linear clusters ranging from 7.2 to 26.5 Å in length in Figure 4 agree most closely with the experimental spectra in the post-white line and low EXAFS regions, showing little difference for different cluster sizes. The longer linear clusters also give reasonable results in the shoulder region. However, all linear clusters display significant discrepancies relative to the experiment in the pre-edge and white line regions. The Frechet FOM supports this qualitative assessment, showing that the linear clusters provide the best quantitative results in the post-white line region and near-best results in the low EXAFS region. Despite the visual discrepancies in the white line region, the long linear clusters provide the best results in these regions of all clusters studied. Similar agreement and discrepancies with respect to experiment are observed for the planar clusters with side length of 7.2–26.5 Å, but with slightly better agreement in the pre-edge and shoulder region (Figure 4b). Thus, these clusters provide the best overall agreement for all shapes and sizes simulated here. Simulated spectra for cubic clusters begin to resemble those of simulated and experimental bulk CaO, notably in the post-white line and EXAFS regions of the spectra (Figure 4c). Despite providing the best agreement in the pre-edge region, discussed in more detail below, they show an overall less satisfactory agreement than spectra simulated based on the linear and planar structure. Thus, if present, they can be regarded as a minority species. In summary, this analysis suggests that the simulated spectra generated from larger linear and planar structures agree best with experimental results. With these data, it is not possible to definitively conclude which of these structures with low dimensionality is the majority species present in these catalysts because each produces spectra with similarities to experimental results in different spectral regions. Without privileging specific spectral regions in the assessment of structures, the results suggest that a mixture of these ultrasmall supported one- and two-dimensional CaO clusters is likely.

**Figure 5:**
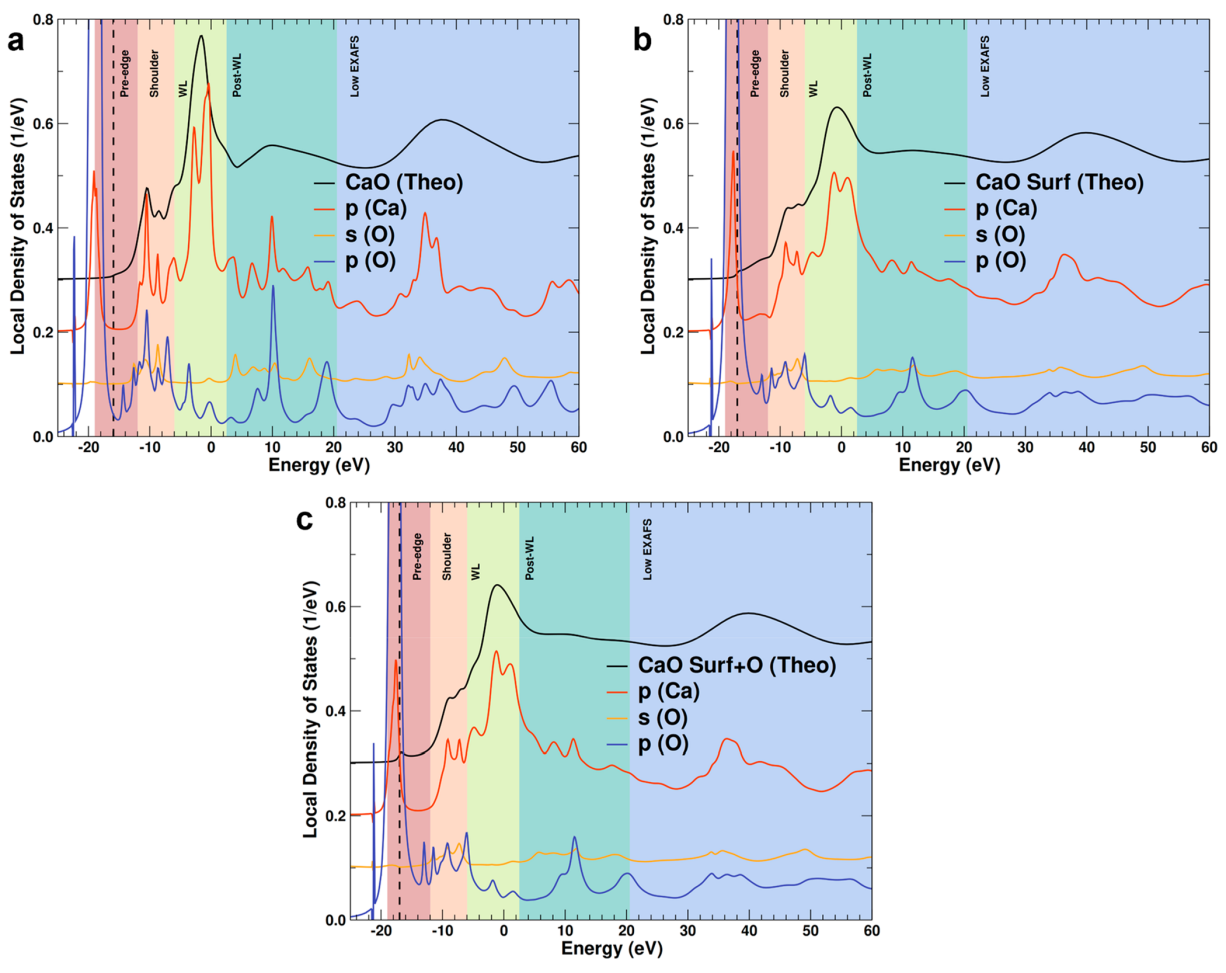
Projected local density of states for (a) bulk CaO, (b) surface Ca atoms, and (c) oxygen-terminated surface Ca atom. The dashed line represents the Fermi level.

The angular momentum decomposed local density of state (LDOS), shown in Figure 5, helps to qualitatively identify which electronic transitions contribute to each region of the spectra. Given the computational cost of the LDOS simulations, here we use Ca on the surface as a proxy for the behavior of undercoordinated atoms in the clusters. In bulk CaO, the white line is dominated by transitions to Ca 4p states, in agreement with selection rules and previous assignments.^50^ The shoulder and post-white line regions show clear Ca p–O p hybridization, reflecting oxygen ligation. No electronic transition in the pre-edge region is observed. The LDOS for a surface Ca atom demonstrates hybridization with oxygen p states in the shoulder and post-white line regions, as observed for bulk CaO, but also with much lower intensity. In the LDOS for surface Ca atoms, a pre-edge feature arising from Ca p states is found to exist, likely due to empty p states formed from reduced coordination. When the surface Ca atom is fully coordinated by the addition of a single oxygen atom (Figure 5c), the pre-edge feature disappears, likely due to the conversion and shift to higher energies of the empty p-like surface state into a σ* state associated with the new CaO bond. This pre-edge peak has been reported to be the Ca 1s to 3d transition of asymmetric clusters,^39,51^ an assumption taken from the interpretation of 3d transition metals. However, Figure 5 demonstrates that the pre-edge can result from the Ca p states of undercoordinated surface Ca atoms. Simulations of the 1s to 3d transition required the activation of quadrupole transitions. Figure S16 deconvolutes the influence of the 1s to p and 1s to d transitions on the LDOS. The 1s to d transition has a negligible contribution to the rise of the pre-edge peak in the undercoordinated surface Ca atom. Geometric distortions around the central absorbing Ca atom do not increase the intensity of the 1s to d transition (Figure S16b,d,f). The LDOS and the weight of the quadrupole transitions suggest the pre-edge peak results from the empty Ca p states in undercoordinated Ca for the unrelaxed structures simulated here. This analysis of the pre-edge peak must be carefully reconciled with the fact that the EXAFS best fit is obtained with fully O-coordinated Ca. Therefore, it is possible that the too large pre-edge peaks observed for the linear and planar clusters compared to the experimental result are due to the presence of too many undercoordinated atoms in these systems, and better results might be obtained by partially capping them. Altogether, computational analysis of XANES data suggests that catalysts with 1 mol % Ca or less are composed of ultrasmall supported CaO clusters organized into linear and planar atomic structures with monolayer thickness.

### 3.4 Initial CaO Cluster Speciation Affects CO_2_-OCM Performance

Evaluation of the CO_2_-OCM performance of these catalysts, reported previously, showed that selectivity to methane coupling was significantly greater for catalysts with less than 2 mol % Ca compared with those with higher Ca loadings.^21^ In the low-loading regime, increased Ca loading was associated with increased C_2_ product yields. The further observation that the rate of methane coupling normalized by Ca loading was similar among low-loading samples suggested that in the low-loading regime, C_2_ production rate increases with an increase in the number of interface sites between CaO and ZnO. At and above 2 mol % Ca loading, further Ca addition only slows the production rate of C_2_ species due to the formation of an inactive bulk CaO phase. Characterization by adsorption FTIR revealed that the low loading catalysts were electronically distinct from bulk CaO, likely due to increased interaction with ZnO.^21^

The XAS results reported here validate the previous hypothesis that the Ca species present in the low-loading catalysts are highly dispersed as ultrasmall clusters. As the Ca loading increases, the cluster size becomes slightly larger. Increasing the size of linear and planar CaO structures decreases the amount of the exposed ZnO surface, blocking sites unselective to coupling and creating new, highly selective interfacial sites. For an equivalent number of atoms, the fraction of CaO–ZnO interfacial atomic sites is considerably higher for linear and planar structures than that for the cubic structure. The undercoordinated Ca species present in the clusters, as suggested to exist by the presence of the pre-edge feature, may also serve as selective methane coupling sites independent of the ZnO interaction.^52,53^

### 3.5 Relating CaO Structure to N_2_O-OCM Performance and Kinetics

**Figure 6:**
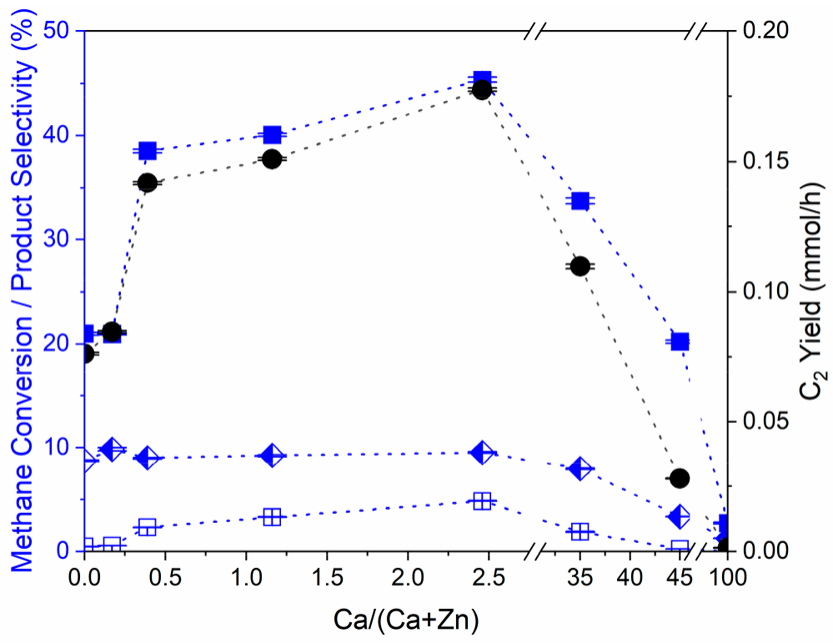
Effects of Ca loading on methane conversion (blue diamond, left half solid), C_2_ selectivity (blue solid square), C_3,4_ selectivity (blue open square), and C_2_ yield (black solid circle). Reaction conditions: 550 °C; 0.75 g of catalyst; 13.3 mL min^–1^ total gas flow rate with *p*_CH_4__ = 0.23 atm, *p*_N_2_O_ = 0.45 atm, *p*_Ar_ = 0.32 atm; average steady-state values with error calculated with 95% confidence interval. All Ca loadings are reported in molar concentrations of metal cations, as determined by ICP-OES (Table S2). All reactant conversions and product yields are reported in Table S9. Dotted lines are included to guide the eye.

The Ca/ZnO catalysts with varying Ca loadings were evaluated in this study for their N_2_O-assisted methane coupling (N_2_O-OCM) activity, which complements our prior catalysis results for CO_2_-OCM.^21^ Hydrocarbon products observed include ethane, ethylene, propane, propylene, and trace butanes and butenes. As was observed for CO_2_-OCM catalysis, it is revealed that there are two regimes of catalytic activity, defined by Ca loading: below 2 mol % Ca, where C_2_ product selectivity is 39 ± 1%, and higher Ca loadings, where C_2_ product selectivity decreases with Ca loading to only 3% over pure CaO. The transition between these two catalytic regimes correlates with the changes in Ca structure observed by XAS. The catalysts with low-Ca-loading that display higher selectivity to C_2_ products were found to be consistent with the coordination environments of ultrasmall linear and planar CaO clusters, while the less-selective catalysts were consistent with those of bulk CaO phases. The distinct activity in the low-loading regime is reflected in Figure 6 by the increase in both selectivity and yield of C_2_ products between 0.2 and 0.4 mol % Ca loading; this is the composition range where C_2_ product yields begin to increase during CO_2_-OCM.^21^ At the lowest Ca loading examined, 0.2 mol %, the presence of Ca on the surface had only a minor influence on N_2_O-OCM catalysis. However, further addition of linear and planar CaO structures can quickly cover the catalyst surface with selective sites, reflected in the strong influence of loading on performance. Normalizing the activity shown in Figure 6 of the supported cluster catalysts by the mass of Ca, as determined by ICP, results in normalized C_2_ product yields of 98 and 35 mmol/h/g_Ca_ over the 1 mol % Ca/ZnO and 0.4 mol % Ca/ZnO catalysts, respectively. The low dimensionality of the CaO clusters implies that nearly all of the Ca atoms are surface-exposed. The normalized C_2_ product yield decreases as the cluster sizes become larger, which correlates with the decreasing concentration of undercoordinated Ca atoms associated with increasing cluster size, reflected by the decreasing pre-edge feature. C_3+_ yields shown in Table S9 trend with those of the C_2_ products. These higher hydrocarbons are likely secondary products formed by further coupling of C_2_ species.^54^ Catalysts with ≥35 mol % Ca where large, bulk CaO particles exist have reduced methane coupling performance with increasing loading. Partial or complete combustion to CO_*x*_ is favored with increasing loading (Table S9) as the catalyst surface becomes more covered by bulk CaO particles; activity resembles that of pure CaO at the highest loadings.^52,55^ The trends of selectivity and product yields for N_2_O-OCM are strongly correlated, suggesting that methane oxidation is in direct competition with methane coupling (presumably with the release of methyl radicals^56^) over these catalysts, an observation that is in contrast with findings from CO_2_-OCM catalysis, where bulk CaO phases were inactive due to stable carbonate formation.

Direct comparisons of reaction outcomes between CO_2_-OCM^21^ with N_2_O-OCM over Ca/ZnO catalysts are complicated by their differingreaction conditions. In general, lower methane coupling selectivities were observed during N_2_O-OCM than those during CO_2_-OCM. However, much higher reactor temperatures are necessary for CO_2_-OCM due to the high degree of stable CaCO_3_ formation as well as the high endothermicity associated with that reaction. At present, this trade-off prevents a conclusive determination of which reaction system achieved better methane coupling performance. Instead, the data illustrate the versatility of the Ca/ZnO catalyst system and the strong influence of calcium active site structure on methane coupling performance in multiple reactive systems.

**Figure 7:**
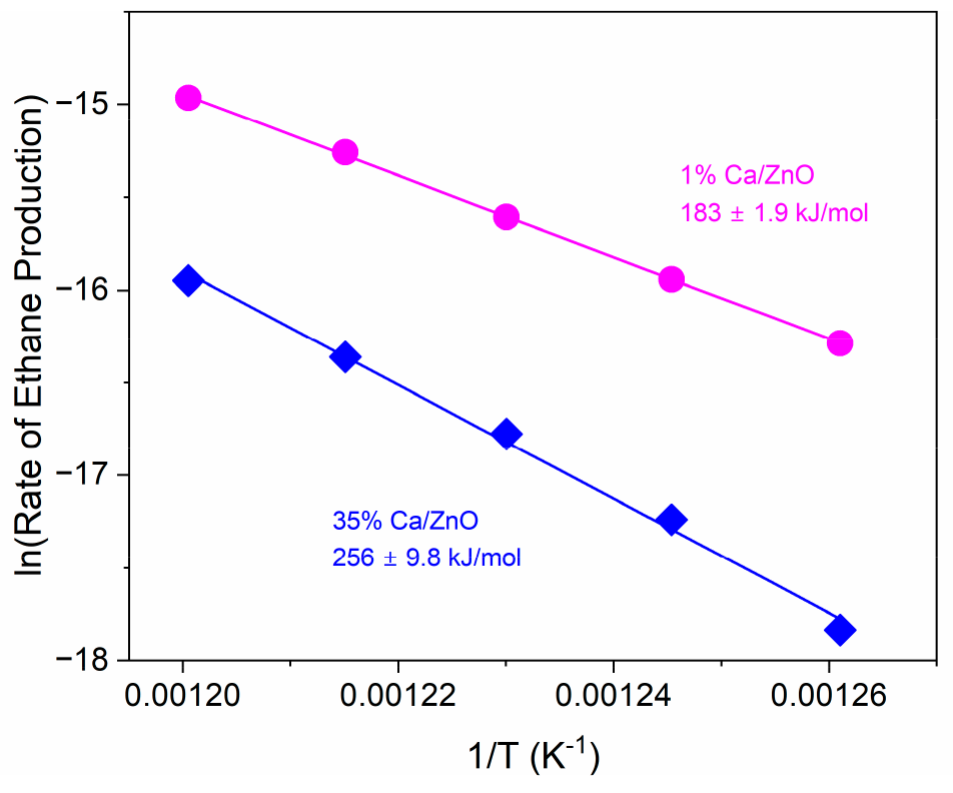
Comparison of activation energies for methane coupling to ethanes over 1 mol % Ca/ZnO and 35 mol % Ca/ZnO: 200 mg of catalyst, total flow of 30 mL/min, CH_4_:N_2_O:Ar feed ratio of 1:2:1, temperature range of 520–560 °C. Error is calculated from a linear fit.

Analyses of results from XAS and reactor studies establish that there are distinct regimes of both CaO structure and catalytic activity as Ca loading is varied. The impact of the two structural and catalytic regimes—methane coupling-selective small CaO clusters and unselective bulk CaO particles—on reaction kinetics was further studied by comparing the activation energies of 1 mol % Ca/ZnO and 35 mol % Ca/ZnO (Figure 7). Plots of reactant conversion and product yields versus inverse space velocity in Figure S17 confirm that the reactor is operating in the differential regime. The activation energies for ethane formation, the primary product of methane coupling, are 183 ± 1.9 and 256 ± 9.8 kJ/mol for 1 mol % Ca/ZnO and 35 mol % Ca/ZnO, respectively. The activation energy of 183 kJ/mol is in good agreement with that previously reported for a similar reactant composition over Li/MgO, where oxygen incorporation from N_2_O to the catalyst surface was determined to be the rate-determining step.^54^ The significantly larger *E*_a_ of 35 mol % Ca/ZnO suggests either a different active site structure, a change in the rate-determining step, or both. The initial CaO structures differ based on the XAS results here, with undercoordinated Ca sites in 1–2D clusters only existing in the low-Ca-loading catalysts. For basic metal oxide catalysts, the presence of CO_2_ from reaction products can inhibit methane coupling, shifting the *E*_a_ to higher energy due to the formation of carbonate species.^53,54,57^ Carbonate can poison CaO active sites and will require higher temperatures to desorb on the high-Ca-loading Ca/ZnO catalysts.^21^ Therefore, the higher *E*_a_ over the catalyst with a bulk CaO phase is due to a different active site structure that is associated with a different rate-determining step than catalysis with the structure present in 1 mol % Ca/ZnO.

Bulk CaO is very effective at N_2_O decomposition to N_2_ and O_2_.^58−60^ N_2_O decomposition rates are completely unaffected by the presence of methane over CaO,^61^ supporting our finding that bulk CaO does not perform N_2_O-OCM but facilitates N_2_O decomposition and secondary oxidation reactions. During N_2_O decomposition, surface oxygen species are formed as intermediates in the reaction path leading to desorbed O_2_.^62,63^ Various adsorbed oxygen species have been found responsible for the overoxidation of methane and hydrocarbon products during OCM.^64,65^ The surface species formed on CaO during N_2_O decomposition can interact strongly with the hydrocarbons present, leading to eventual oxidation. Gas-phase oxidation to CO_*x*_ species can also occur via CH_3_• oxidation by molecular O_2_.^66^ Therefore, the ethane observed to be generated over 35 mol % Ca/ZnO is hypothesized to be a result of the reaction occurring at interfacial sites between CaO and ZnO.

The generation of CO_2_ leads to the formation of a stable CaCO_3_ phase at the reaction temperature.^53^ While CaO is still the dominant phase in Ca/ZnO catalysts after N_2_O-OCM, the carbonate phase can be observed by XRD on all catalysts with ≥1 mol % Ca content after N_2_O-OCM (Figure S18). However, pure CaO is converted mostly to carbonate. CaCO_3_ is a poor N_2_O decomposition catalyst.^67^Figure S19 compares the activity of CaO and CaCO_3_. The conversion of N_2_O over CaO is initially 100% and gradually decreases with time on stream as carbonate surface species form. The steady-state C_2_ product selectivity over CaO and CaCO_3_ are 3% and 14%, respectively. Calcium carbonate formation on bulk CaO therefore does not significantly improve the methane coupling performance but inhibits the decomposition of N_2_O decomposition. Higher C_2_ product selectivities are observed over ZnO-supported CaO clusters than over bulk CaO or CaCO_3_ catalysts. While carbonate deposition likely occurs on supported CaO clusters during reaction, these catalysts maintain their enhanced methane coupling performance relative to bulk CaO. Neither the rate of carbonate deposition nor any potential *in situ* evolution of CaO during reaction is studied here. All results presented herein indicate the presence of a relationship between the microstructural environment of ZnO-supported Ca in the synthesized catalysts and activity toward methane coupling with alternative oxidants N_2_O and CO_2_.

## 4 Conclusion

This work investigates the local physical and electronic structures of Ca in ZnO-supported CaO soft-oxidant-assisted methane coupling catalysts through Ca K-edge XANES and EXAFS. XANES results have been interpreted through simulated spectra derived from *ab initio* multiple scattering calculations (FEFF), which elucidate the structural correlates of sites that are active for methane coupling. Results show that below 2 mol % Ca loading, the Ca sites are associated with a coordination environment consistent with CaO clusters organized as one- and two-dimensional structures with approximately one atomic layer thickness. The Ca–Ca coordination numbers derived from EXAFS data suggest that the size of these clusters is approximately 7.2–26.5 Å. The simulated spectrum of a surface Ca atom matches well with the experimental spectrum measured for the low-Ca-loading catalysts. An increasing pre-edge intensity with decreasing Ca loading indicates the presence of undercoordinated surface Ca atoms, according to local densities of states calculations. Catalysts containing these low-dimensional CaO clusters yield enhanced CO_2_- and N_2_O-assisted methane coupling activity when compared to catalysts containing CaO particles with bulk properties. Significantly different activation energies for ethane formation over 1 mol % Ca/ZnO and 35 mol % Ca/ZnO catalysts are observed (183 ± 1.9 and 256 ± 9.8 kJ/mol, respectively), which is attributed to observed differences in active site structure and carbonate stability. These results suggest that future investigations of this system should involve *in situ* characterization of the Ca speciation during reactions to probe the significance and influence of carbonate formation on reactivity. These results provide fundamental insights into the active site structure of binary metal oxide catalysts, which contribute to optimization of C_2_ product yields during soft-oxidant-assisted methane coupling.

## Supporting Information Available

EXAFS models for *S*_0_^2^ determination; EXAFS data of high-Ca-loading catalysts; rejected EXAFS models of Ca(OH)_2_ and ZnO; wavelet transform analysis; relaxed ZnO-supported CaO cluster structure; simulated XANES spectra of bulk CaO, surface Ca atom, and CaO clusters; Frechet-distance-like error analysis between theory and experiment; summary of N_2_O-OCM catalysis results; XRD characterization of spent catalysts (PDF)
