# Supplementary material for: Impact of Local Structure in Supported CaO Catalysts for Soft-Oxidant-Assisted Methane Coupling Assessed through Ca K-Edge X-ray Absorption Spectroscopy
Source: J Phys Chem C Nanomater Interfaces. 2024 Jan 10;128(3):1165–76. doi: 10.1021/acs.jpcc.3c06527 (PMC10823472; doi:10.1021/acs.jpcc.3c06527)
Supplement: Supplementary file 1 — jp3c06527_si_001.pdf [file jp3c06527_si_001.pdf]

Supplemental Information

Impact of Local Structure in Supported CaO Catalysts for Soft Oxidant-Assisted Methane Coupling Assessed through Ca K-edge X-ray Absorption Spectroscopy

Leah R. Filardi[a], Fernando D. Vila[b], Jiyun Hong[c], Adam S. Hoffman[c], Jorge E. Perez-Aguilar[c], Simon R. Bare[c], Ron C. Runnebaum*[a,d], Coleman X. Kronawitter*[a]

[a]Department of Chemical Engineering, University of California, Davis, CA, 95616 USA
[b]Department of Physics, University of Washington, Seattle, WA, 98195 USA
[c]SSRL, SLAC National Accelerator Laboratory, Menlo Park, CA 94025 USA
[d]Department of Viticulture & Enology, University of California, Davis, CA, 95616 USA

Table of Contents

Table S1. Table of abbreviations……………………………………………………………….. S2
Table S2. Calcium concentration determined from ICP-OES....…………………………...……… S3
Figure S1. Ca K-edge XANES spectra overlayed.………………………………………….……… S4
Figure S2. Fits of 2 mol% Ca/ZnO for S_0^2 determination………………….………..…..………….. S5
Table S3. EXAFS fitting parameters from S_0^2 determination.…….………………...………………… S5
Figure S3. FEFF simulated single-scattering paths of CaO compared to 2 mol% Ca/ZnO…….. S6
Figure S4. EXAFS fitting results of Ca/ZnO catalysts...…………………………………….……….. S7
Figure S5. EXAFS fitting of 0.6 mol % Ca/ZnO as isolated Ca atoms on ZnO………….……..… S8
Table S4. Best-fit EXAFS model of isolated Ca atoms on ZnO.……………………………...….… S8
Figure S6. EXAFS fitting of 0.6 mol% Ca/ZnO as CaO clusters on ZnO……………...……..… S9
Table S5. Best-fit EXAFS model of CaO clusters on ZnO.……………………...…..……….… S9
Figure S7. FEFF simulated k-space of Ca-Ca and Ca-Zn scattering paths……………...……. S10
Figure S8. Continuous Cauchy wavelet transform of EXAFS data…………………………….. S11
Figure S9. Relaxed structure of ZnO-supported CaO cluster………………………………….. S12
Figure S10. EXAFS fitting of 0.6 mol% Ca/ZnO as $Ca(OH)_2$...……………….……………… S13
Table S6. Best-fit EXAFS model of $Ca(OH)_2$……………………………………..………….…. S13
Figure S11. EXAFS fitting of 0.6 mol% Ca/ZnO as a combination of CaO and $Ca(OH)_2$…..… S14
Table S7. Best-fit EXAFS model of a combination of CaO and $Ca(OH)_2$..……………………. S14
Figure S12. Verification of FEFF simulation quality with bulk CaO data…………………….. S15
Figure S13. Simulated XANES of surface & bulk Ca atom compared to experimental spectra. S16
Figure S14. Unrelaxed linear, planar, and cubic CaO cluster structures……………………….. S17
Figure S15. Average simulated XANES spectra of unrelaxed clusters……………………..…….. S18
Table S8. Regional Frechet-distance-like errors between theory and experiment……………... S19
Figure S16. Decomposition of dipole and quadrupole contributions to theoretical spectra.…… S20
Table S9. Summary of catalytic N_2O-OCM performance over Ca/ZnO catalysts …………….. S21
Figure S17. Linear-dependence of methane conversion on inverse space velocity …………..…. S22
Figure S18. XRD patterns of catalysts after N_2O-OCM ……………………………………..… S23

Figure S19. N_2O conversion and C_2 selectivity of CaO and $CaCO_3$ …………………………..…. S24

Table S1. Table of Abbreviations

Abbreviation	Full Name
OCM	Oxidative Coupling of Methane
N_2O-OCM	Nitrous Oxide-Assisted Oxidative Coupling of Methane
CO_2-OCM	Carbon Dioxide-Assisted Oxidative Coupling of Methane
TEM	Transmission Electron Microscopy
XRD	X-ray Diffraction
ICP-OES	Inductively Coupled Plasma – Optical Emission Spectroscopy
XAS	X-ray Absorption Spectroscopy
XANES	X-ray Absorption Near Edge Structure
EXAFS	Extended X-ray Absorption Fine Structure
CN	Coordination Number
S_0^2	Amplitude Correction Term
σ^2	Disorder Term
ΔE_0	Energy Correction Factor
MSRD	Mean Square Relative Displacement
CCWT	Continuous Cauchy Wavelet Transform
FOM	Figure of Merit
LDOS	Local Density of States

Table S2. Calcium concentration determined from ICP-OES.

**Labeled mol%**	**ICP Quantification mol%**	**ICP Quantification wt%**
0.2%	0.17	0.084
0.4%	0.39	0.193
0.6%	0.58	0.287
1%	1.33	0.659
2%	2.4	1.23
35%	34.5	19.0
45%	45.4	26.0

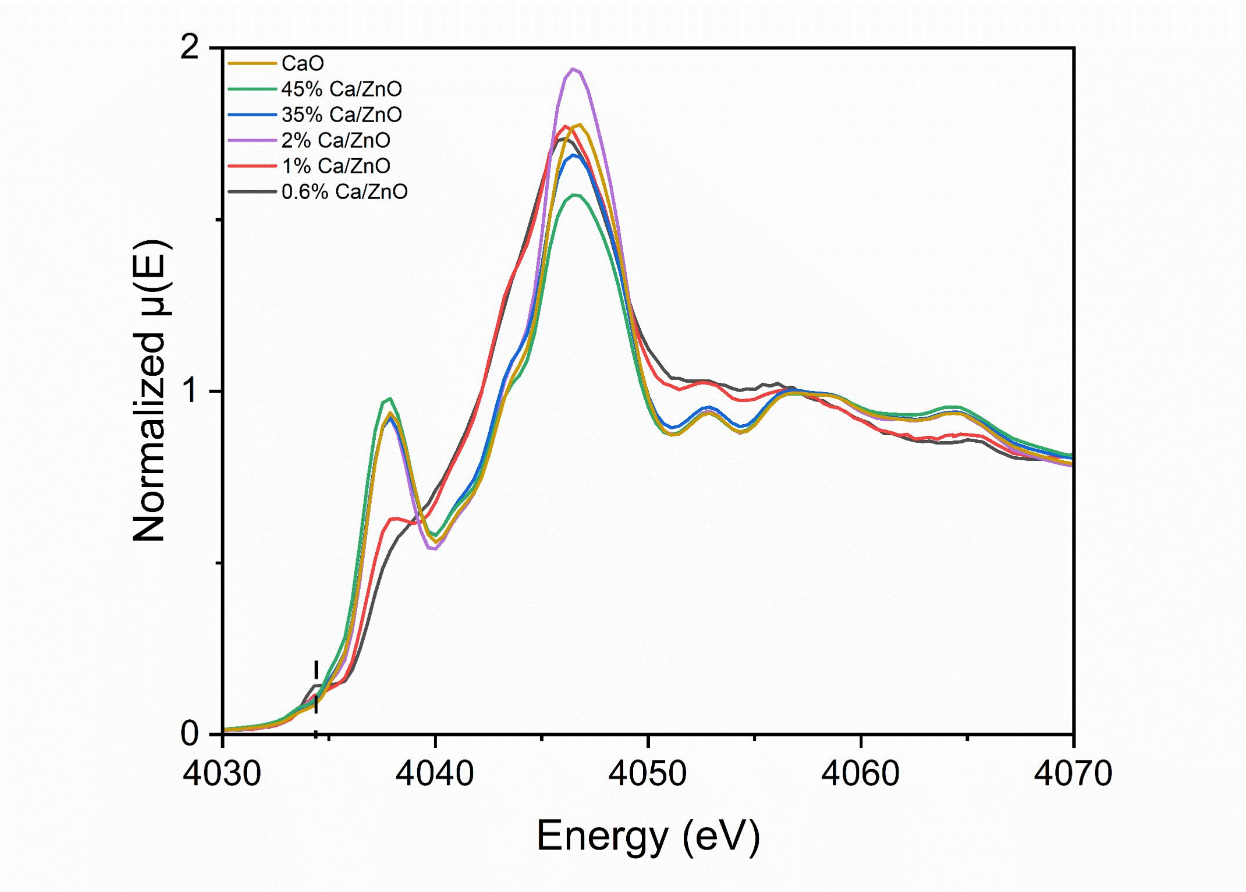

Figure S1. *Ex situ* Ca K-edge XANES spectra characterizing Ca/ZnO catalysts of varying composition collected at room temperature in He. Dashed line to visualize pre-edge feature.

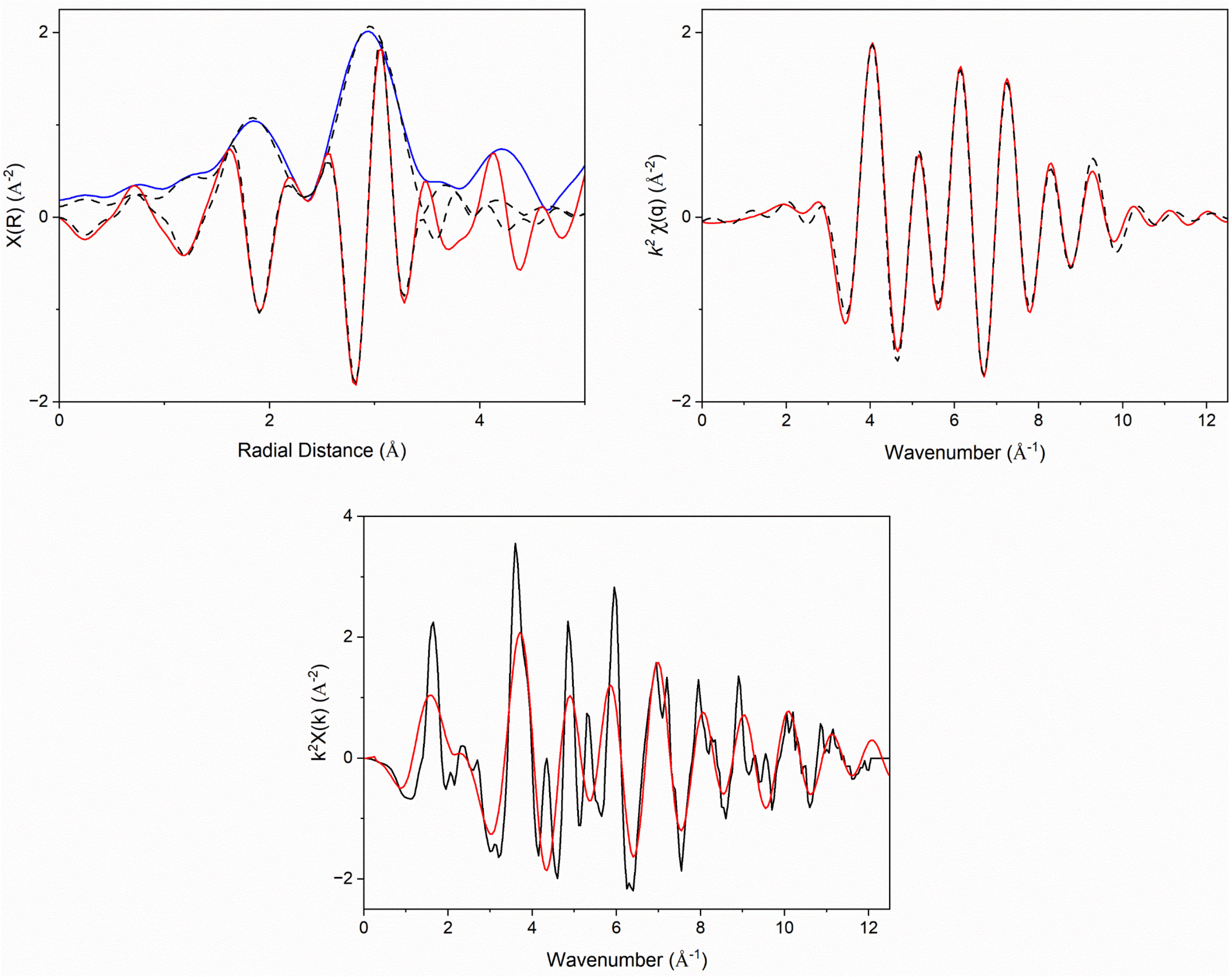

Figure S2. The magnitude (blue) and imaginary (red) portion of the k^2-weighted Fourier transforms of the EXAFS spectra (top left) and q-space function (top right) used to calculate S_0^2 from 2 mol% Ca/ZnO, with fits are shown in dotted lines; k-space (k^2-weighting) data and fits in black and red, respectively (bottom).

Table S3. Best-fit of the EXAFS parameters of 2 mol% Ca/ZnO. Notation: N, coordination number; S_0^2, amplitude correction term; ΔE_0, energy correction factor; R, scattering path length; σ^2, disorder term. Values without error bounds were held constant. A k-range of 3.4-9.1 $Å^{-1}$ and R-range of 1.0-3.5 Å were used.

**Reference**	**Path**	**N**	**R (Å)**	**σ^2 x 10^3 ($Å^2$)**	**ΔE_0 (eV)**	**R-Factor**	**S_0^2**
2% Ca/ZnO	Ca-O	6	2.39 ± 0.01	5.2 ± 2.0	0.3 ± 0.6	0.0044	0.73
	Ca-Ca	12	3.41 ± 0.01	4.6 ± 1.0			

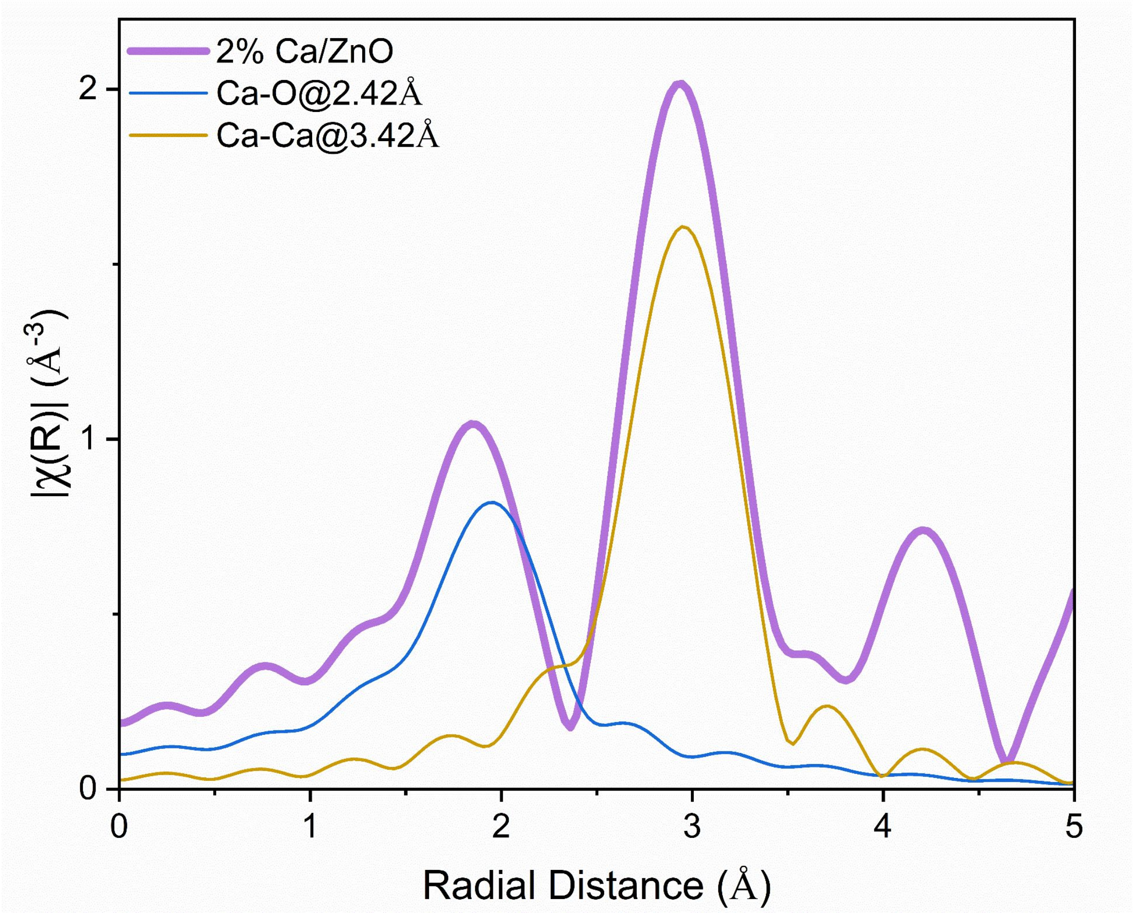

Figure S3. Magnitude of the Fourier transform of the first two phase-uncorrected single-scattering paths of CaO: Ca-O at bond distance of 2.42 Å and Ca-Ca at 3.42 Å, simulated with FEFF6. The comparison of the simulated paths to 2 mol% Ca/ZnO confidently describes the first 2 peaks of the data.

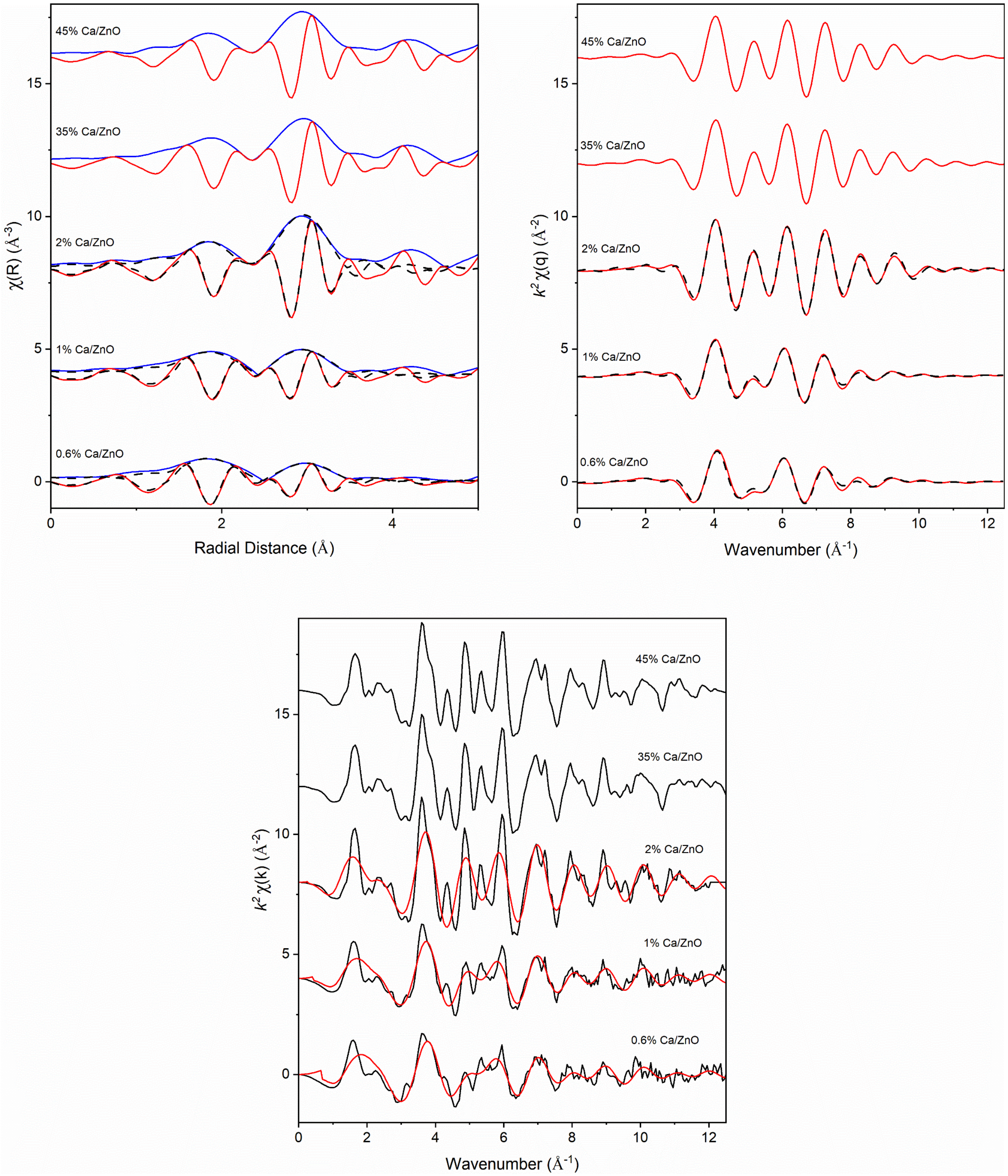

Figure S4. The magnitude and imaginary portion of the k^2-weighted Fourier transforms of the EXAFS spectra (top left) are shown using solid blue and red lines, respectively and q-space data (top right) shown in red, where fits are shown with dotted lines; k-space (k^2-weighting) data and fits in black and red, respectively (bottom).

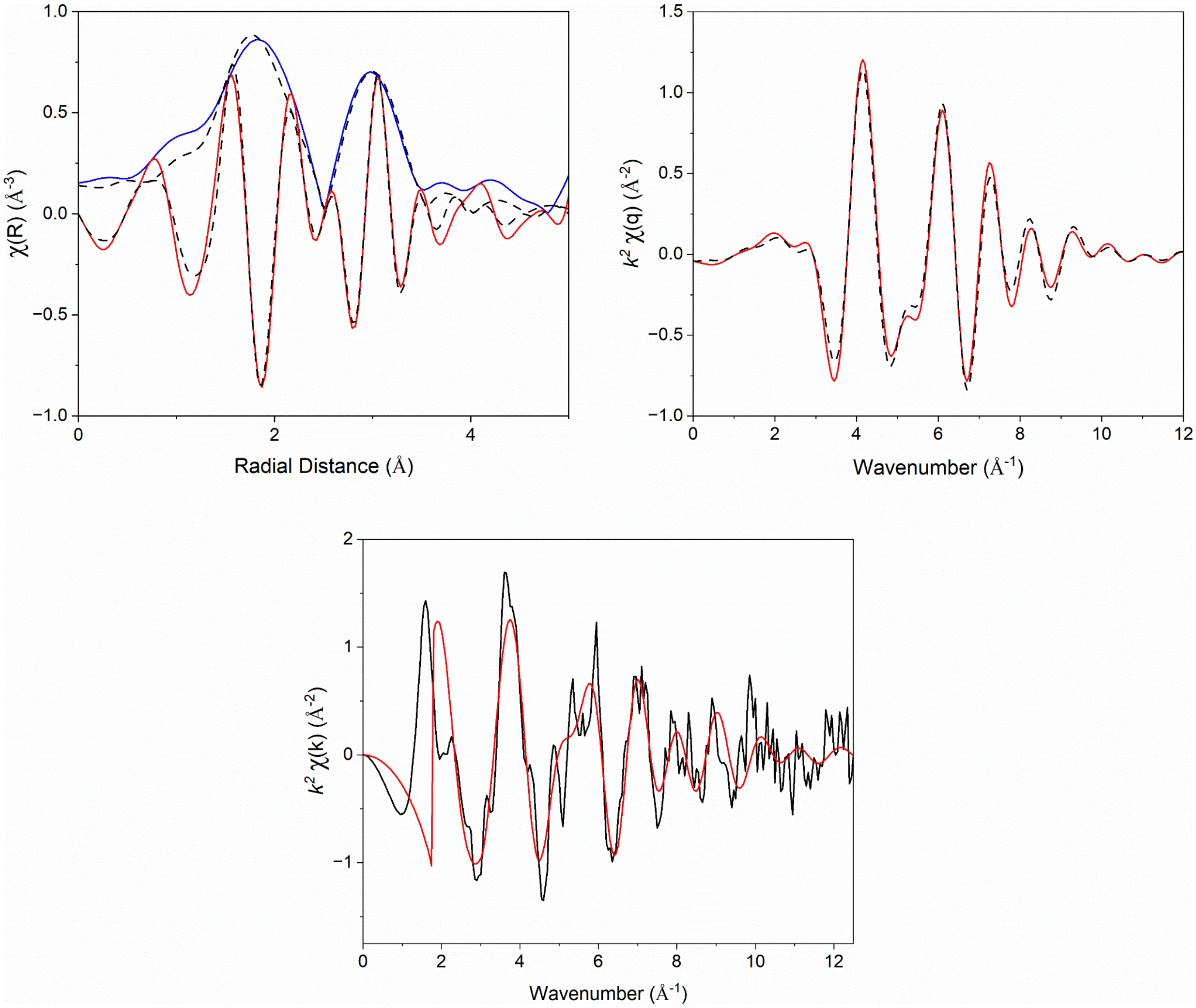

Figure S5. The magnitude and imaginary portion of the k^2-weighted Fourier transforms of the EXAFS spectra (top left) are shown using solid blue and red lines, respectively, and q-space data (top right) of the 0.6 mol% Ca/ZnO sample shown in red, where fits using a model of an isolated calcium atom on ZnO are shown with dotted lines; k-space (k^2-weighting) data and fits in black and red, respectively (bottom).

Table S4. Best-fit of the EXAFS parameters. Notation: CN, coordination number; S_0^2, amplitude correction term; ΔE_0, energy correction factor; R, scattering path length; σ^2, disorder term. Values without error bounds were held constant. A k-range of 3.4-9.1 Å^{-1} and R-range of 1.0-3.5 Å were used. Both paths were generated from a cif file replacing a single Zn atom within ZnO with Ca.

**Path**	**CN**	**R (Å)**	**σ^2 x 10^3 (Å^2)**	**ΔE_0 (eV)**	**R-Factor**	**S_0^2**
Ca-O	6	2.23 ± 0.02	10.1 ± 1.7	11.9 ± 1.3	0.0104	0.73
Ca-Zn	9.1 ± 3.1	3.25 ± 0.01	12.8 ± 3.7			

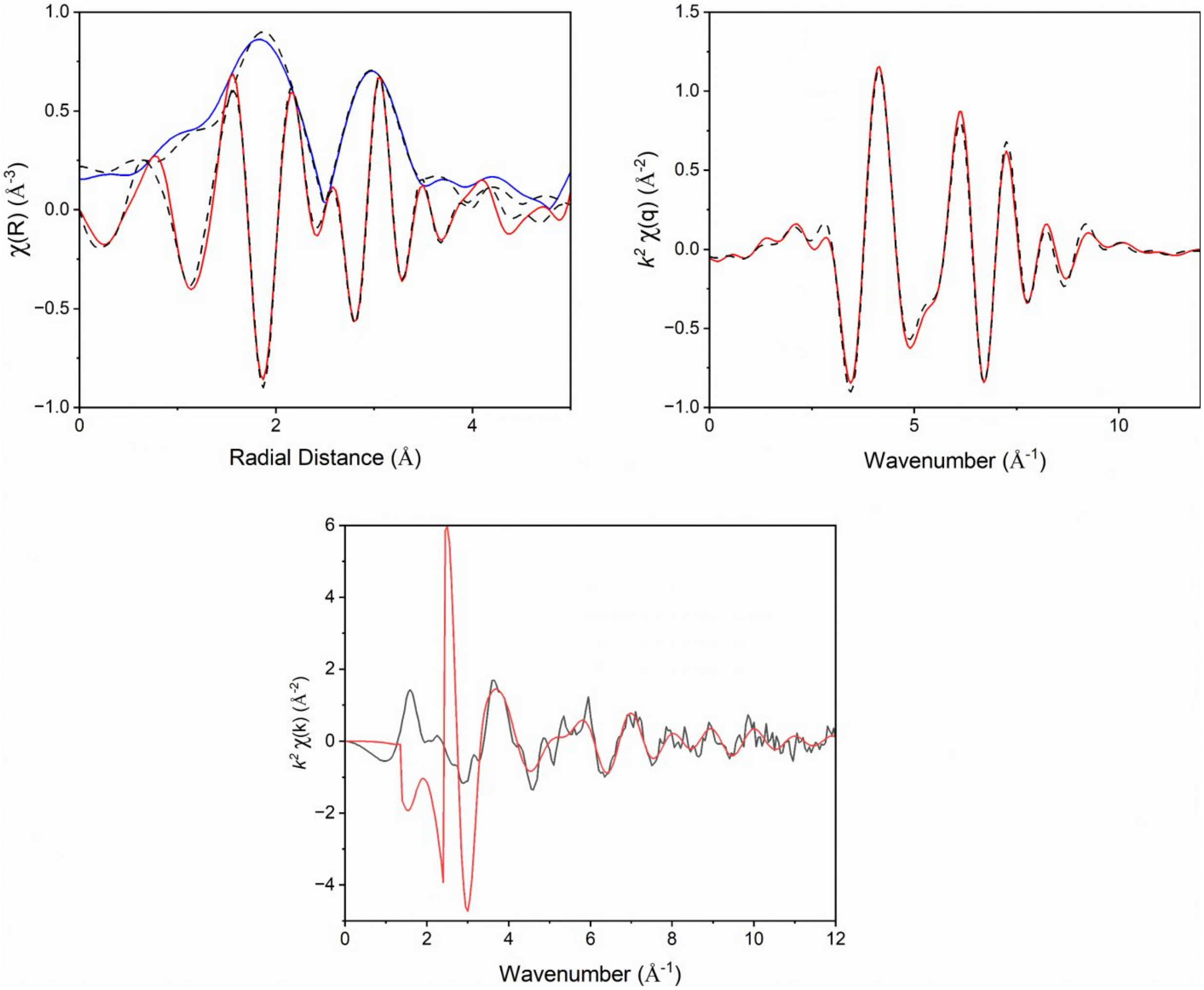

Figure S6. The magnitude and imaginary portion of the k^2-weighted Fourier transforms of the EXAFS spectra (top left) are shown using solid blue and red lines, respectively, and q-space data (top right) of the 0.6 mol% Ca/ZnO sample are shown in red, where fits using a model of calcium oxide cluster on ZnO are shown with dotted lines; k-space (k^2-weighting) data and fits in black and red, respectively (bottom).

Table S5. Best-fit of the EXAFS parameters. Notation: CN, coordination number; S_0^2, amplitude correction term; ΔE_0, energy correction factor; R, scattering path length; σ^2, disorder term. Values without error bounds were held constant. A k-range of 3.4-9.1 Å^{-1} and R-range of 1.0-4.0 Å were used.

**Path**	**CN**	**R (Å)**	**σ^2 x 10^3 (Å^2)**	**ΔE_0 (eV)**	**R-Factor**	**S_0^2**
Ca-O	6	2.37 ± 0.1	8.2 ± 6.5	1.2 ± 6.5	0.02130	0.73
Ca-Ca	2.3 ± 2.2	3.43 ± 0.1	4.6			
Ca-Zn	14.5 ± 110.3	3.47 ± 0.6	64.3 ± 156.6	-9.3 ± 81.4		

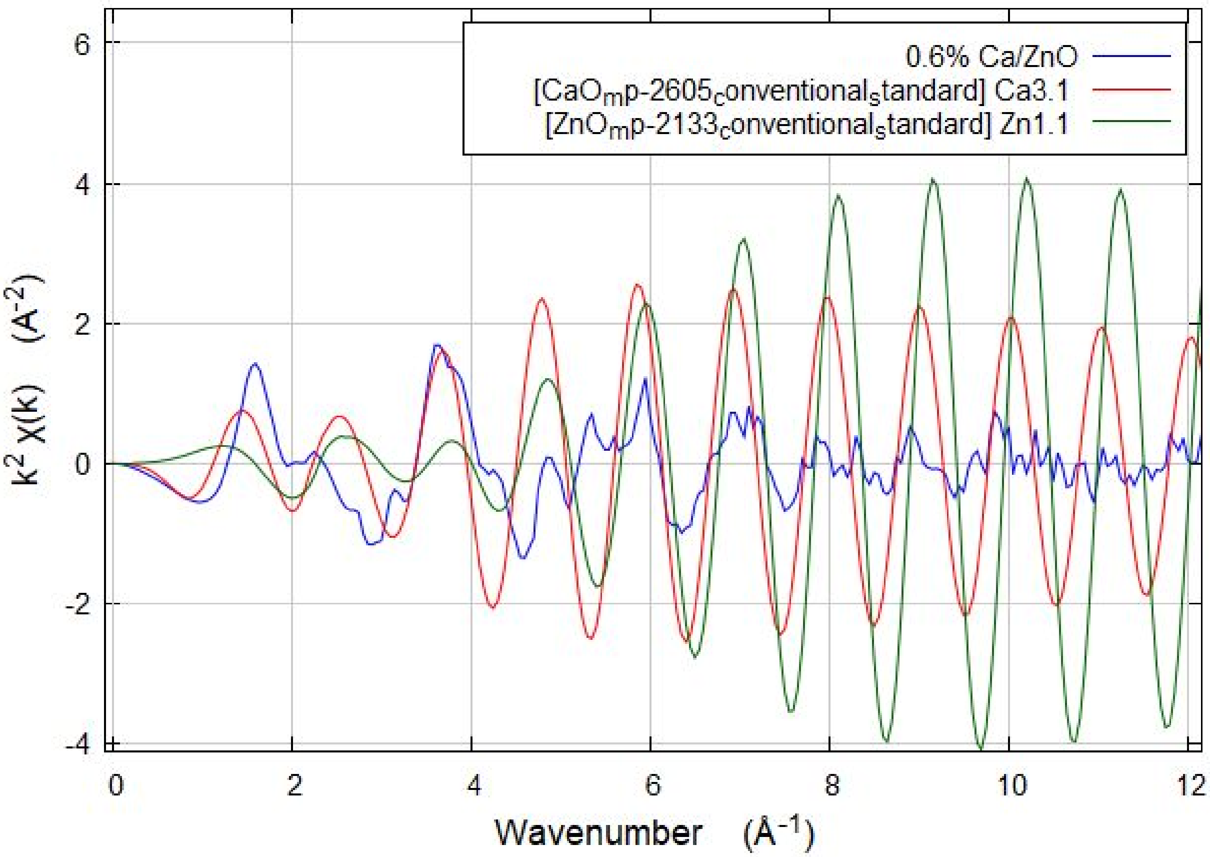

Figure S7. Simulated k-space oscillations of second nearest neighbor Ca and Zn atom scattering paths compared to experimental data.

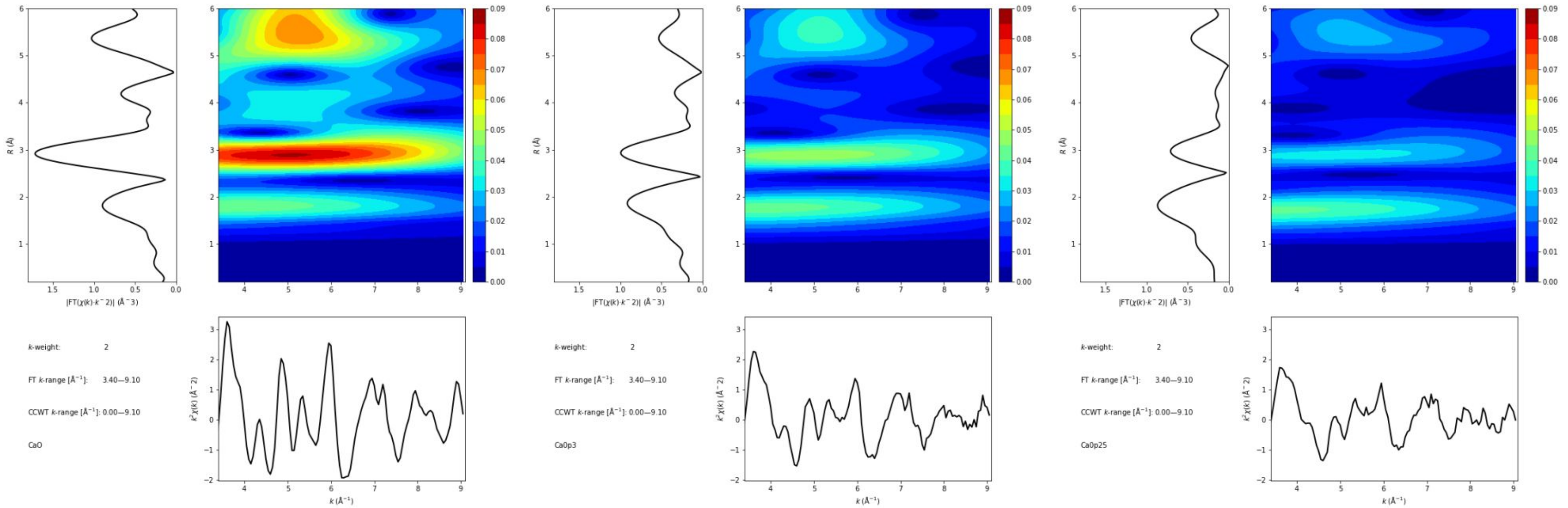

Figure S8. Continuous Cauchy wavelet transform of k^2-weighted (left) CaO, (middle) 1% Ca/ZnO, and (right) 0.6% Ca/ZnO EXAFS spectra reveals that the R-space peak centered around 3 Å does not contain any detectable influence from a heavier scattering element.

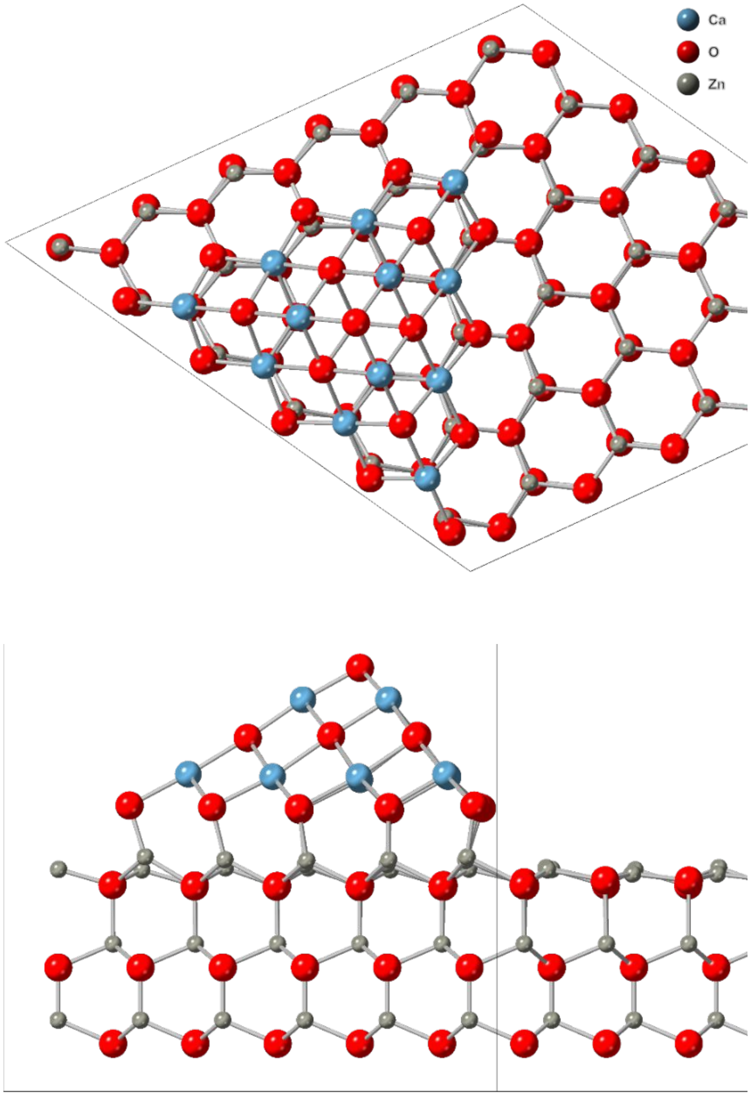

Figure S9. Top-view (top) and side-view (bottom) of a representative, DFT-relaxed, ZnO-supported CaO cluster cleaved along the O-exposed (111) plane to create optimal epitaxial contact with the support. The optimized cluster has an average number of Ca-Ca near-neighbors of 4.6 ± 1.2, similar to that observed in the EXAFS, making it relevant for comparison to the experimental results. The final optimized structure expands laterally with respect to the initial one to release virtually all the epitaxial contraction, resulting in an average bond distance of 3.40 Å, nearly identical to the bulk CaO distance of 3.39 Å at the same level of theory. This results in a bond strain of 1.00 ± 0.01. Moreover, the Ca-Ca shell distribution is narrow, with a static mean square relative displacement (MSRD) of 3.6×10^{-3} Å^2. The Ca-Zn shell has an average bond distance of 3.43 Å, thus lying in the range of the Ca-Ca one. The average Ca-Zn number of near-neighbors is 3.0 ± 0.0. However, the Ca-Zn path static disorder is very large, with an MSRD of 22.4×10^{-3} Å^2. This probably results in the Ca-Zn signal being completely overwhelmed by the Ca-Ca one, thus making the support invisible in the EXAFS.

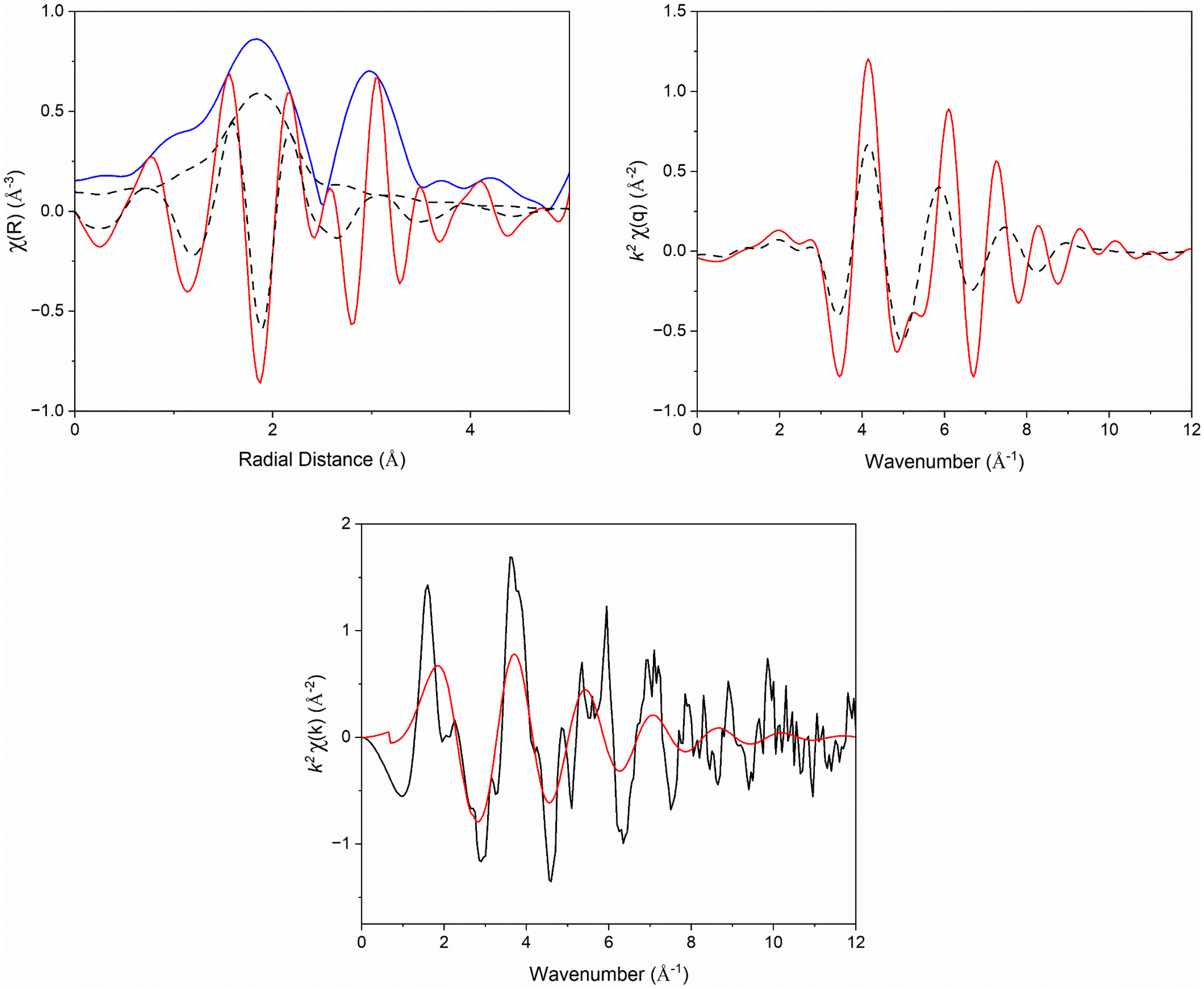

Figure S10. The magnitude and imaginary portion of the k^2-weighted Fourier transforms of the EXAFS spectra (top left) are shown using solid blue and red lines, respectively, and q-space data (top right) of the 0.6 mol% Ca/ZnO sample shown in red, where fits using a model of pure calcium hydroxide are shown with dotted lines; k-space (k^2-weighting) data and fits in black and red, respectively (bottom).

Table S6. Best-fit of the EXAFS parameters. Notation: CN, coordination number; S_0^2, amplitude correction term; ΔE_0, energy correction factor; R, scattering path length; σ^2, disorder term. Values without error bounds were held constant. A k-range of 3.4-9.1 Å^{-1} and R-range of 1.0-3.5 Å were used.

**Path**	**CN**	**R (Å)**	**σ^2 x 10^3 (Å^2)**	**ΔE_0 (eV)**	**R-Factor**	**S_0^2**
Ca-O	6	2.37 ± 0.3	12.1 ± 17.4	1.8 ± 25.6	0.5453	0.73
Ca-Ca	0.0 ± 16.8	3.63	10.8 ± 183472			

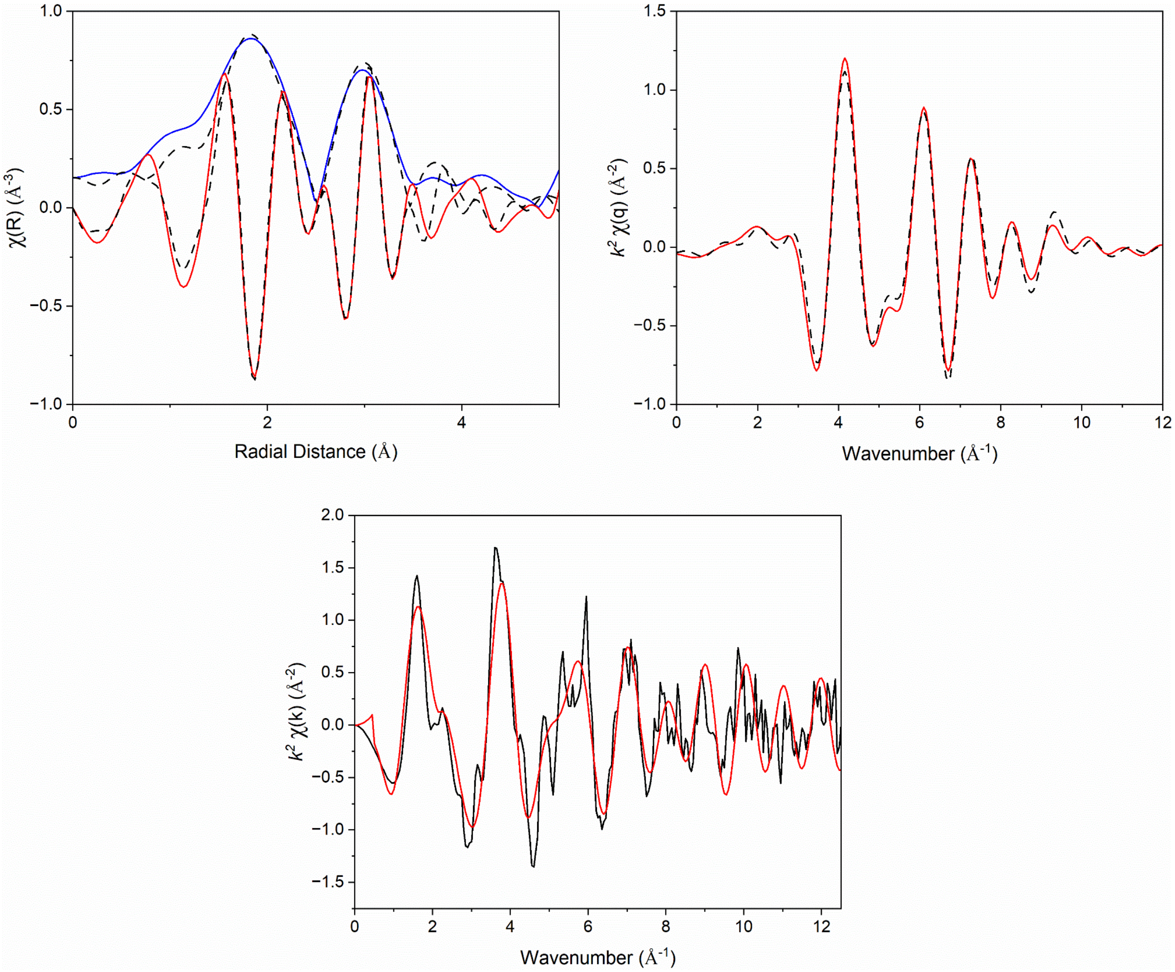

Figure S11. The magnitude and imaginary portion of the k^2-weighted Fourier transforms of the EXAFS spectra (top left) are shown using solid blue and red lines, respectively, and q-space data (top right) of the 0.6 mol% Ca/ZnO sample shown in red, where fits using a model of both calcium hydroxide and calcium oxide are shown with dotted lines; k-space (k^2-weighting) data and fits in black and red, respectively (bottom).

Table S7. Best-fit of the EXAFS parameters. Notation: CN, coordination number; S_0^2, amplitude correction term; ΔE_0, energy correction factor; R, scattering path length; σ^2, disorder term. Values without error bounds were held constant. A k-range of 3.4-9.1 Å^{-1} and R-range of 1.0-3.5 Å were used.

**Path**	**CN**	**R (Å)**	**σ^2 x 10^3 (Å^2)**	**ΔE_0 (eV)**	**R-Factor**	**S_0^2**
Ca-O	6	2.36 ± 0.1	6.9 ± 7.7	0.9 ± 9.6	0.0088	0.73
Ca-Ca (oxide)	11.5 ± 34.6	3.47 ± 0.1	4.6			
Ca-Ca (hydroxide)	11.1 ± 43.3	3.63	4.5 ± 1.1	-6.9 ± 14.5		

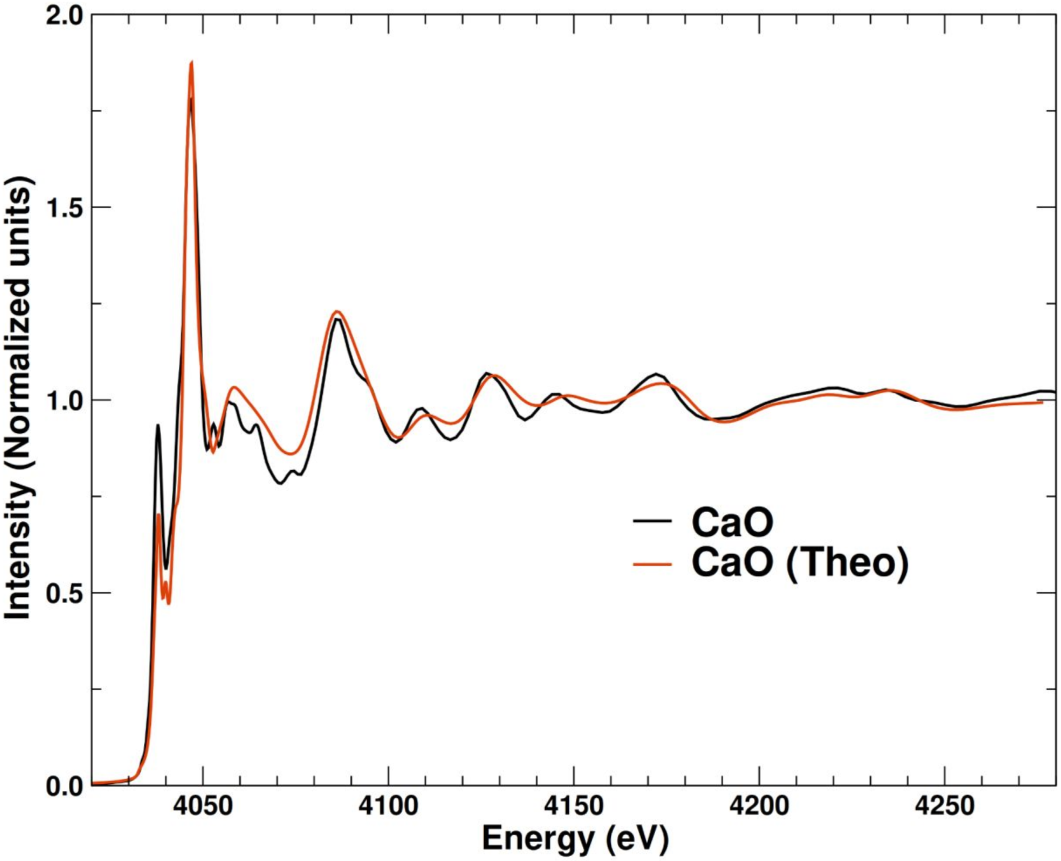

Figure S12. Comparison of experimentally measured Ca K-edge XANES spectrum of CaO reference to simulated XANES spectrum of a Ca atom from within the bulk of a large CaO structure.

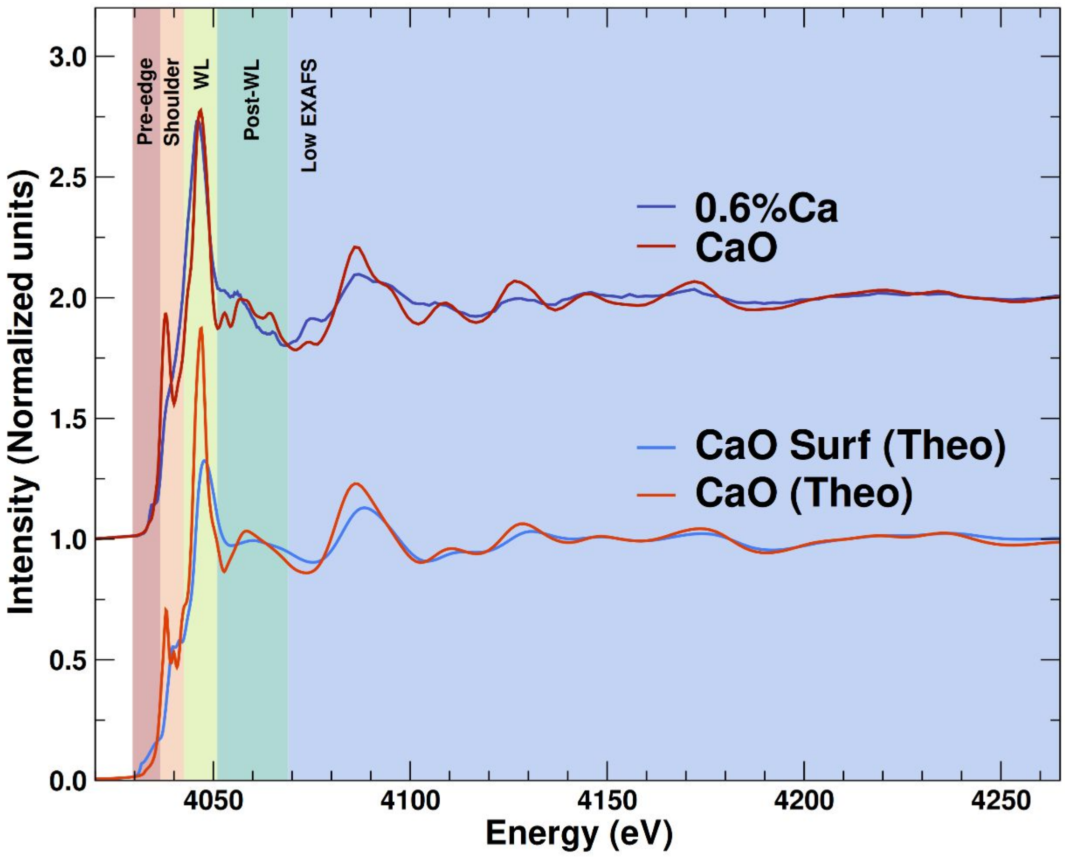

Figure S13. Comparison of experimental spectra of 0.6 mol% Ca/ZnO and bulk CaO reference to simulated spectra of a surface Ca atom of a CaO slab and a bulk Ca atom.

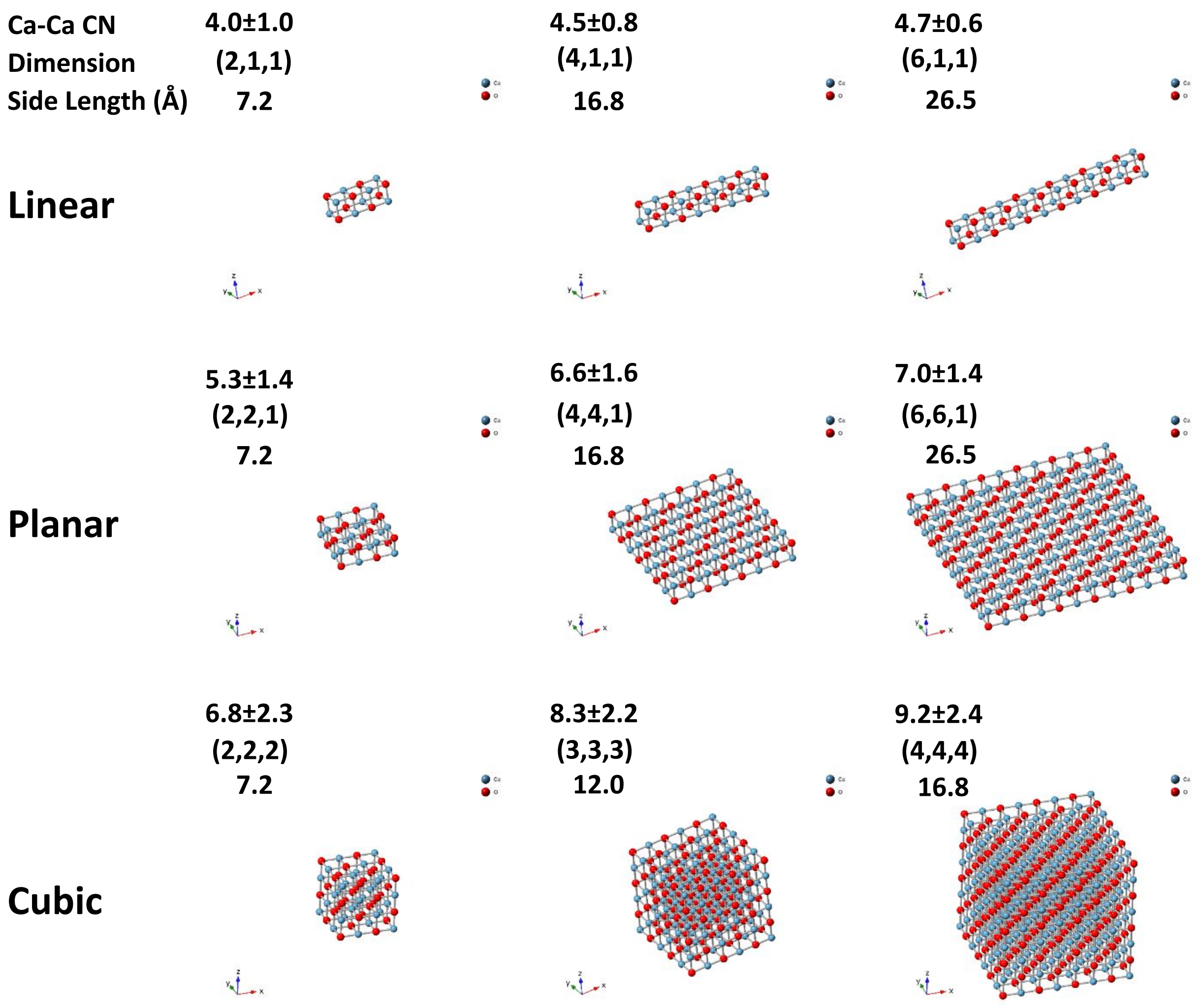

Figure S14. CaO structures of varying size and morphology used to simulate Ca K-edge spectra

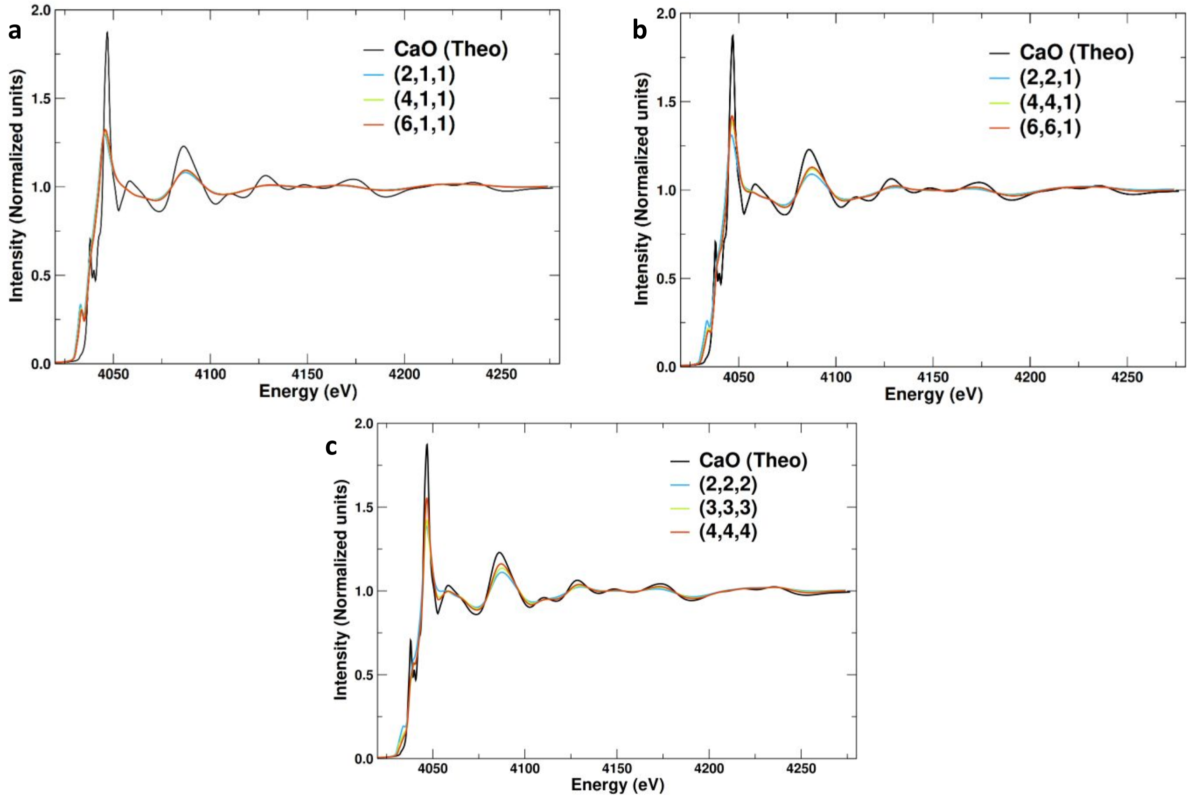

Figure S15. Comparison between the simulated XANES spectra of bulk CaO and varying sizes of (a) linear, (b) planar, and (c) and cubic structures.

Table S8. Total and regional ΔXANES matching Frechet-distance-like errors between theory and experiment. The Frechet figure of merit (FOM) is defined as: Given a reference function f_1 and a target f_2 represented by the curve segments with coordinates $\{x_{1i}, y_{1i}\}$ and $\{x_{1i}, y_{1i}\}$, we define the vector $\vec{d}_{1i2} = \{d_{1i2j}\}$ of distances from point i in f_1 to any point in f_2. With these distances we can define the FOM as $FOM = \sum_i \min\left[\vec{d}_{1i2}\right]/L$, where the normalization factor L is the length of the f_1 curve segment defined as $L = \int_{x_{11}}^{x_{1n}} dx_1 \sqrt{1 + \left(\frac{\partial f_1}{\partial x_1}\right)^2}$.

NP	Pre-edge	Shoulder	WL	Post-WL	Low EXAFS	Total
**Ln_01_01_01**	5.09	6.72	5.07	0.74	0.34	0.82
**Ln_02_01_01**	3.72	4.59	4.19	0.70	0.33	0.69
**Ln_03_01_01**	3.22	3.29	4.03	**0.68**	0.31	0.62
**Ln_04_01_01**	3.23	3.93	3.79	0.70	0.31	0.63
**Ln_05_01_01**	3.05	3.63	3.62	0.69	0.31	0.61
**Ln_06_01_01**	2.96	3.51	**3.56**	0.69	0.31	0.60
**Pl_02_02_01**	2.65	2.82	3.85	0.70	0.35	0.62
**Pl_03_03_01**	2.10	2.54	3.63	**0.68**	0.33	0.57
**Pl_04_04_01**	1.80	2.42	3.82	0.71	**0.30**	0.55
**Pl_05_05_01**	1.67	2.40	3.84	0.71	**0.30**	0.54
**Pl_06_06_01**	1.56	**2.39**	3.75	0.72	**0.30**	**0.53**
**Cu_02_02_02**	1.73	2.59	4.20	0.70	0.35	0.60
**Cu_03_03_03**	1.18	2.92	4.71	0.85	0.33	0.61
**Cu_04_04_04**	**0.83**	3.02	3.99	0.85	0.36	0.60

NP	Pre-edge	Shoulder	WL	Post-WL	Low EXAFS	Total
**CaO Expt vs Theo**	0.11	0.84	0.51	0.28	0.10	0.15

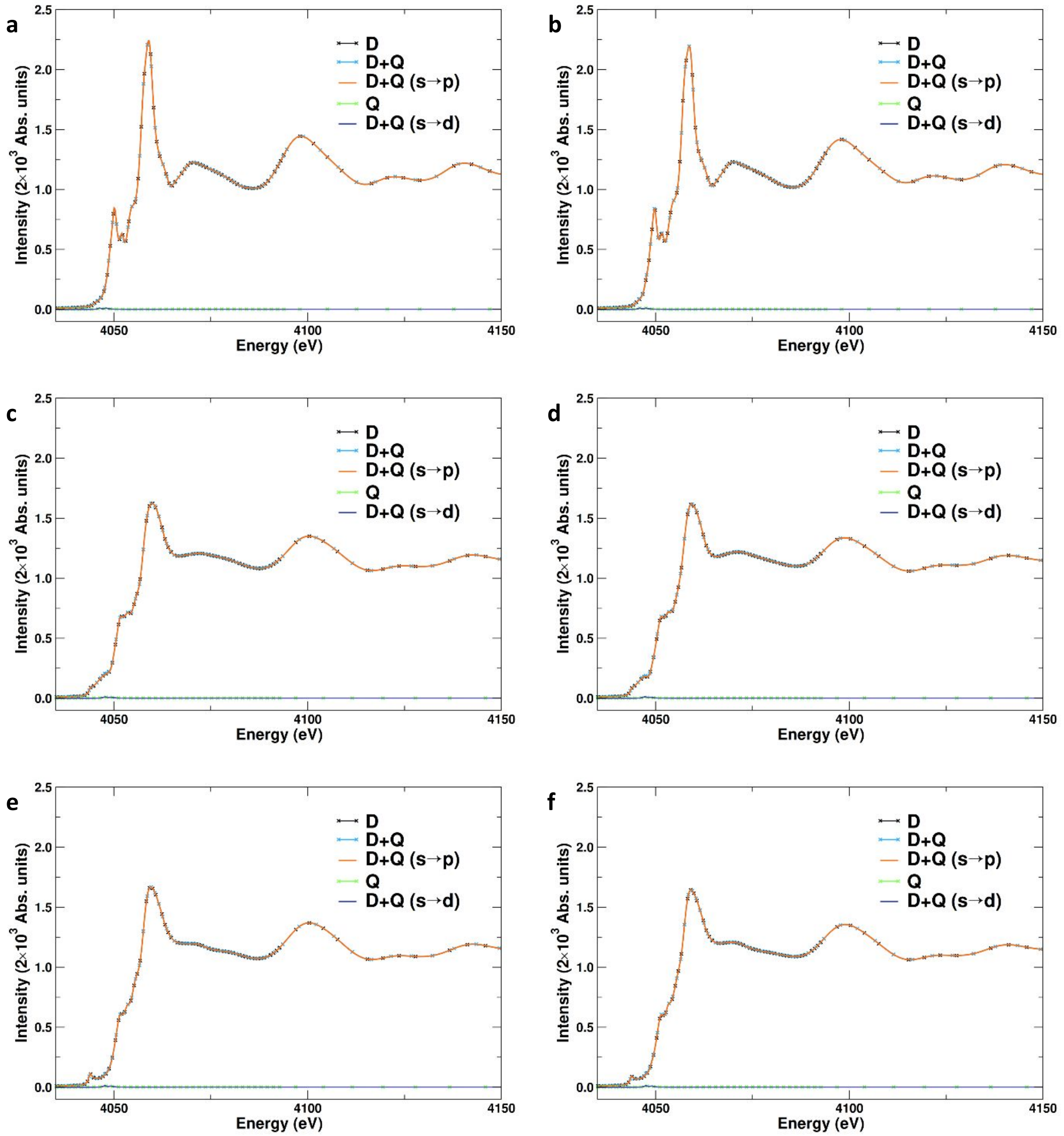

Figure S16. Decomposition of the dipole and dipole+quadrupole contributions to the theoretical spectra of (a-b) bulk CaO, (c-d) surface Ca atom, and (e-f) oxygen-terminated surface Ca atom into their s→p and s→d components (a, c, e) without and (b, d, f) with a distortion applied to the central Ca atom of +0.1 Å along the z-axis.

Table S9. Summary of catalytic performance over the range of Ca loadings.

Ca Loading	CH_4 Conversion (%)	N_2O Conversion (%)	C_2H_6 Yield x10^2 (mmol/h)	C_2H_4 Yield x10^2 (mmol/h)	C_3H_8 Yield x10^3 (mmol/h)	C_3H_6 Yield x10^4 (mmol/h)	C_4 Yield x10^4 (mmol/h)	CO Yield x10^2 (mmol/h)	CO_2 Yield x10^1 (mmol/h)
ZnO	8.7 ± 0.1	11.9 ± 0.2	6.3 ± 0.1	1.3 ± 0.1	1.1 ± 0.1	0.5 ± 0.1	0	0.4 ± 0.1	5.7 ± 0.1
0.2%	9.8 ± 0.2	13.8 ± 0.3	6.4 ± 0.1	2.1 ± 0.1	1.4 ± 0.1	0.8 ± 0.1	0.2 ± 0.1	0.5 ± 0.1	6.3 ± 0.1
0.4%	9.0 ± 0.1	9.4 ± 0.1	11.1 ± 0.1	3.1 ± 0.1	5.3 ± 0.1	3.8 ± 0.1	3.1 ± 0.1	0.9 ± 0.1	4.2 ± 0.1
1%	9.2 ± 0.1	9.2 ± 0.1	12.2 ± 0.1	2.9 ± 0.1	7.5 ± 0.1	8.0 ± 0.1	5.2 ± 0.3	1.5 ± 0.1	4.1 ± 0.1
2%	9.5 ± 0.1	7.6 ± 0.1	14.6 ±0.1	3.2 ± 0.1	10.7 ± 0.1	18.6 ± 0.1	7.4 ± 0.1	4.0 ± 0.3	3.5 ± 0.1
35%	8.0 ± 0.1	8.2 ± 0.8	9.4 ± 0.1	1.5 ± 0.1	3.5 ± 0.1	5.4 ± 0.2	1.4 ± 0.1	7.0 ± 0.2	3.5 ± 0.1
45%	3.4 ± 0.1	5.2 ± 0.1	2.8 ± 0.1	0.01 ± 0.1	0.2 ± 0.1	0	0	0.1 ± 0.1	2.2 ± 0.1
CaO	1.2 ± 0.1	11.4 ± 0.4	0.1 ± 0.1	0.01 ± 0.1	0	0	0	0.5 ± 0.1	1.0 ± 0.1
$CaCO_3$	1.2 ± 0.4	1.3 ± 0.2	0.5 ± 0.1	0.02 ± 0.1	0	0	0	1.9 ± 0.2	0.5 ± 0.1

Reaction conditions: 550 °C; 0.75 g catalyst; 13.3 mL min^{-1} total gas flow rate with p_{CH_4} = 0.23 atm, p_{N_2O} = 0.45 atm, p_{Ar} = 0.32 atm; average steady state values with error calculated with 95% confidence interval. All Ca loadings are reported in molar concentration of metal cations, as determined by ICP-MS (Table S2).

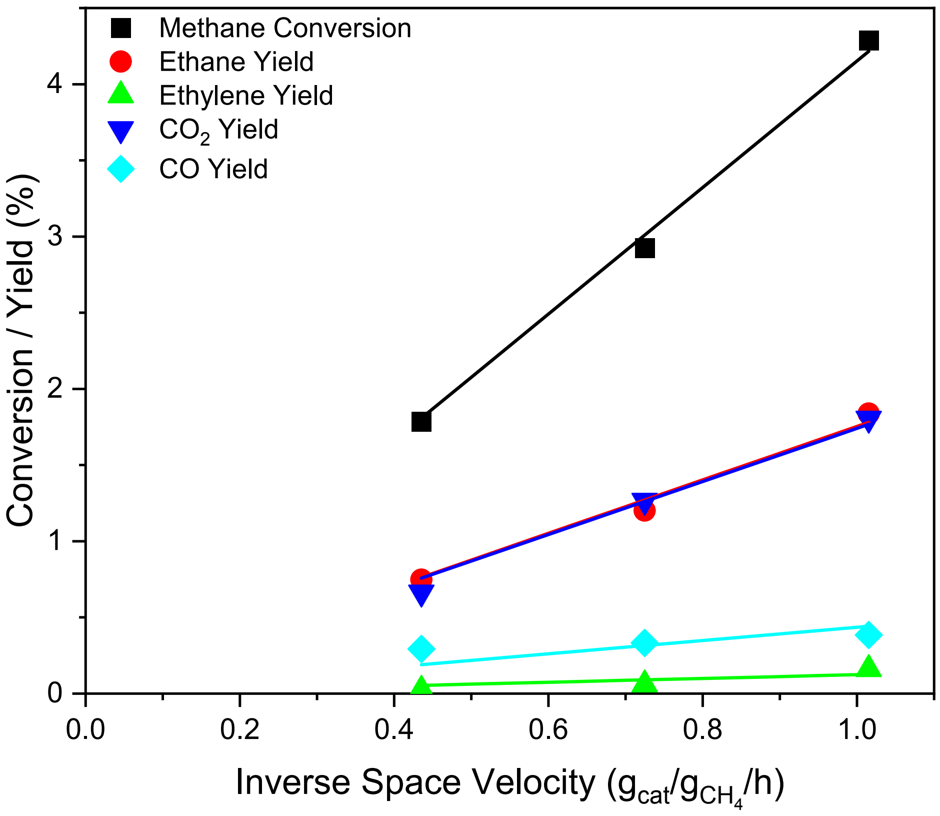

Figure S17. Inverse space velocity of reactant conversion and product rates confirming differential regime during steady state N_2O-OCM at 560 °C, 43.75 mL/min total flow, CH_4:N_2O:Ar feed ratio of 1:3:1, varying catalyst mass (0.15 g, 0.25 g, 0.35 g).

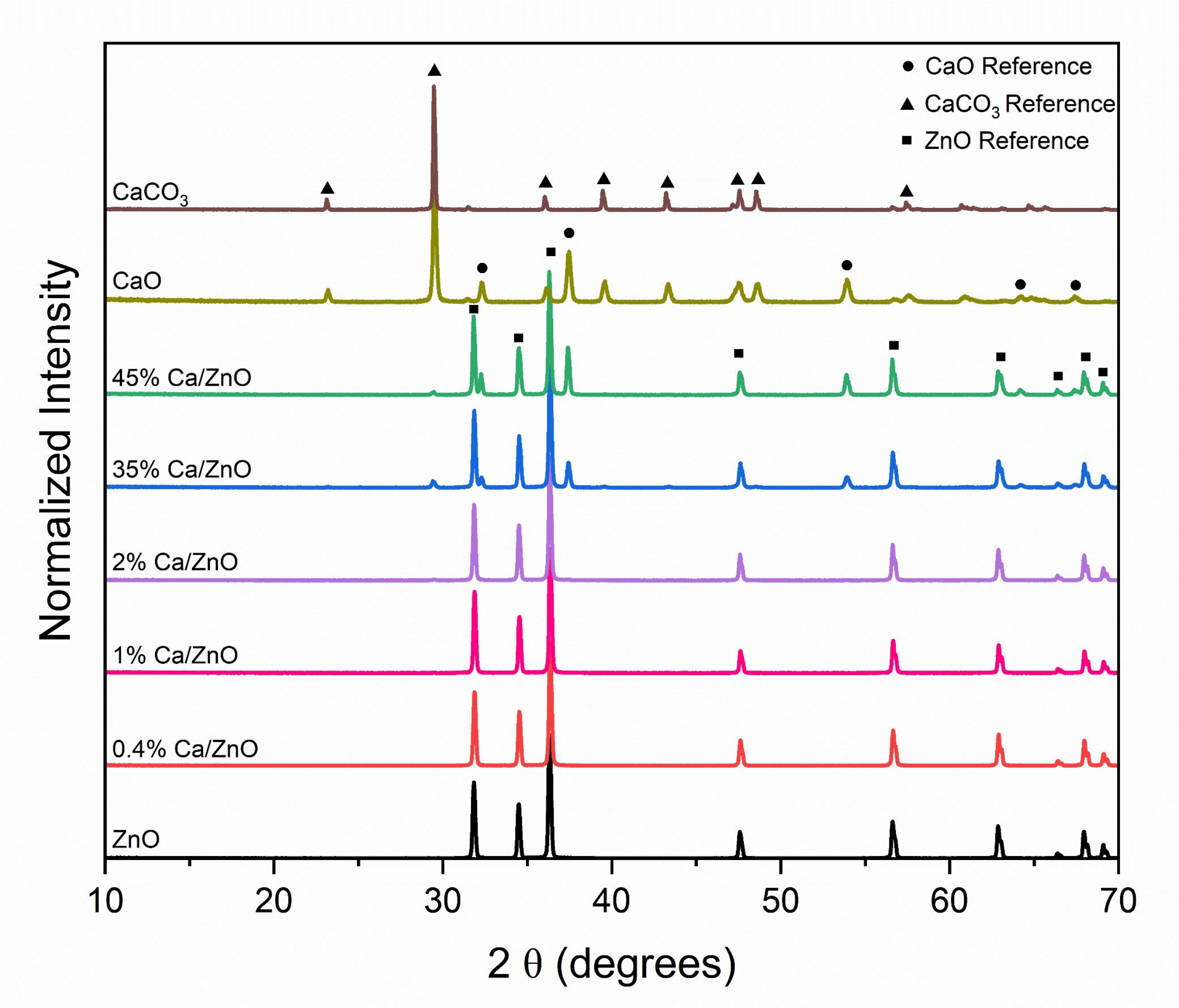

Figure S18. X-ray diffractograms of catalysts after N_2O-OCM. Diffractograms were collected using a Bruker D8 Advanced Diffractometer with Cu Kα radiation. Powder catalysts were sieved through a 304 stainless steel wire cloth disc with a mesh size of 200 to ensure a random distribution of exposed facets, then adhered to silica sample holders with Dow Corning high vacuum grease.

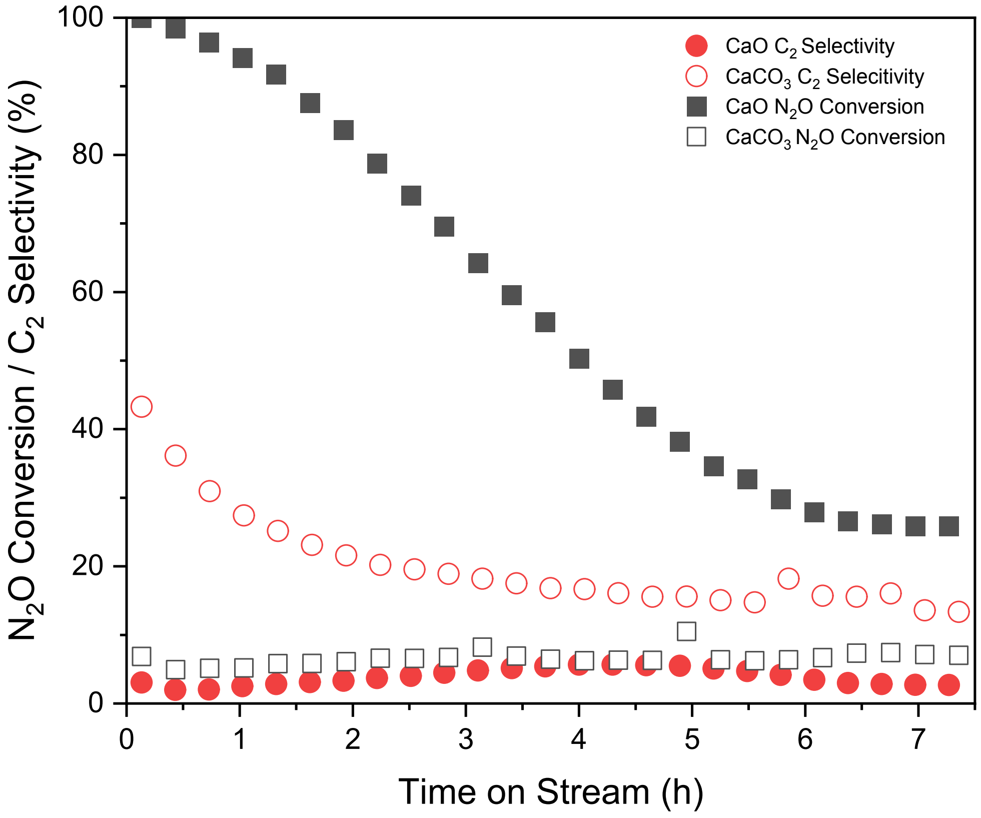

Figure S19. Reactivity of CaO and $CaCO_3$ over the course of N_2O-OCM. Reaction conditions: 550 °C; 0.75 g catalyst; 13.3 mL min^{-1} total gas flow rate with p_{CH_4} = 0.23 atm, p_{N_2O} = 0.45 atm, p_{Ar} = 0.32 atm.
